# A Structural-Based Strategy for Recognition of Transcription Factor Binding Sites

**DOI:** 10.1371/journal.pone.0052460

**Published:** 2013-01-08

**Authors:** Beisi Xu, Dustin E. Schones, Yongmei Wang, Haojun Liang, Guohui Li

**Affiliations:** 1 Laboratory of Molecular Modeling and Design, State Key Laboratory of Molecular Reaction Dynamics, Dalian Institute of Chemical Physics, The Chinese Academy of Sciences, Dalian, Liaoning, China; 2 Department of Microbiology, Immunology and Biochemistry, University of Tennessee Health Science Center, Memphis, Tennessee, United States of America; 3 Center for Integrative and Translational Genomics, University of Tennessee Health Science Center, Memphis, Tennessee, United States of America; 4 Department of Cancer Biology, Beckman Research Institute, City of Hope, Duarte, California, United States of America; 5 Department of Chemistry, University of Memphis, Memphis, Tennessee, United States of America; 6 Department of Polymer Science and Engineering, University of Science and Technology of China, Hefei, Anhui, China; Indian Institute of Science, India

## Abstract

Scanning through genomes for potential transcription factor binding sites (TFBSs) is becoming increasingly important in this post-genomic era. The position weight matrix (PWM) is the standard representation of TFBSs utilized when scanning through sequences for potential binding sites. However, many transcription factor (TF) motifs are short and highly degenerate, and methods utilizing PWMs to scan for sites are plagued by false positives. Furthermore, many important TFs do not have well-characterized PWMs, making identification of potential binding sites even more difficult. One approach to the identification of sites for these TFs has been to use the 3D structure of the TF to predict the DNA structure around the TF and then to generate a PWM from the predicted 3D complex structure. However, this approach is dependent on the similarity of the predicted structure to the native structure. We introduce here a novel approach to identify TFBSs utilizing structure information that can be applied to TFs without characterized PWMs, as long as a 3D complex structure (TF/DNA) exists. This approach utilizes an energy function that is uniquely trained on each structure. Our approach leads to increased prediction accuracy and robustness compared with those using a more general energy function. The software is freely available upon request.

## Introduction

One of the central challenges of this post-genomic era is to decipher the complex regulatory networks that control gene expression. It is generally true that increased morphological complexity in organisms is not correlated with an increase in gene number, but instead with an increase in regulatory complexity [1]. Gene expression is controlled at various stages involving many factors, including regulatory RNAs, DNA binding proteins and epigenetic modifications such as DNA methylation [2]. One major regulatory component is the binding of transcription factors (TFs) to specific DNA sequences that impart positive or negative control on the transcription of corresponding target genes. Identifying a comprehensive set of binding sites for a given TF is critical in understanding the role of that TF in gene regulatory networks. Despite this importance, the prediction of potential binding sites for many TFs remains challenging [3].

Modern methods for the identification of TFBS mostly use experimental data in combination with computational approaches. The experimental data includes three dimensional (3D) structures of TFs bound to DNA, immunoprecipitated DNA sequences followed by hybridization to microarray chips (ChIP-chip) [4] or massively parallel sequencing (ChIP-Seq) [5], Systematic Evolution of Ligands by EXponential enrichment (SELEX) [6], [7], or protein-binding microarrays (PBMs) [8]. While each of these methods have proven to be useful, they each have their own drawbacks. Methods relying on ChIP experiments are dependent on the availability of high-quality antibodies against given proteins of interests. For SELEX, one must be careful to avoid over-selection because factors can bind *in vivo* to biologically important medium- or low-affinity loci as well. A major problem with PBMs is that in vitro affinities may differ from in vivo binding dependent on the current state of the chromatin environment. However, methods utilizing 3D structures of TFs do not have these limitations. With an increasing number of solved structures of protein/DNA complexes in Protein Data Bank (PDB) [9], it is becoming more common to identify TFBSs using structural information of solved protein/DNA complexes [10]–[29].

The general strategy of structural based prediction of TFBSs includes two primary steps. First, one starts with a structure of a TF bound with its cognate DNA sequence. This can be an experimentally determined structure or one computationally predicted through homology modeling and/or docking. The use of computationally predicted structures is useful when no protein/DNA complex structure information is available for candidate TF. Second, a scoring function is used to evaluate the potential for binding between a TF and its cognate TFBS. The scoring function can be based on molecular mechanics (MM) force fields [25], [26], [30], [31], [32], [33], [34] or knowledge-based potentials [17], [35], [36]. Regardless of which scoring function is used, a PWM is generated for a given TF by comparing the binding site one position at a time to every potential nucleotide. The generated PWM can then be used to scan sequences for putative TFBSs. A good TFBS prediction model should be able to discriminate the real TFBS from other nonsense sequences.

A great variability exists in the scoring functions used for evaluating protein/DNA binding. Mm-based energy functions have the advantage of being physically sound, but may suffer from the transferability of derived parameters between molecules as well as the high computational cost to obtain binding free energy. Knowledge-based energy functions, on the other hand, are derived from statistical analysis of known protein/DNA structures, similar to knowledge-based potentials for protein structure predictions [37]. There is again a great variability in how to derive knowledge-based potentials. Several studies have suggested that an all-atom, distance dependent potential is better for predicting TF/DNA binding specificity [13]. For this reason, we recently extended a previous potential based on the distance-scaled, finite ideal-gas reference (DFIRE) state [38] to account for residue specific atom types for protein/DNA binding [39]. The first application of DFIRE to protein/DNA interaction employed hybrid special atom types; using a total of 19 atom types covering all standard amino acids and bases [18]. In our previous work, we added a volume-fraction correction term to DFIRE to account for the unmixable nature of protein and DNA atoms when residue-specific atom types are used [39]. In addition, we added a low-count correction and a reduced interaction distance cutoff. We will designate the energy function derived in this former paper [39] as vcFIRE.

Given the promise of applying knowledge-based energy functions to the prediction of TFBS sites, we were motivated to explore other modifications that could improve the performance of vcFIRE in this task. Here we considered three modifications to the existing approach in vcFIRE: 1) reweighting of observed atom pairs, 2) new smoothing approaches and 3) a dipolar approximation. These modifications were implemented because traditional energy functions are biased towards larger structures that contain more atoms and the dipolar approximation is useful to capture the properties of dipolar atoms. The performance of the derived energy functions using combinations of the three modified approaches was tested for DNA sequence decoy discrimination, docking decoy discrimination, recovering native base pairs, and prediction of PWMs. After the energy functions were benchmarked, the best performing energy function among them was selected for use in prediction of TFBS for 16 known *Saccharomyces cerevisiae* TFs. Applying the energy function with a fixed DNA backbone to the upstream sequences of yeast open reading frames (ORFs) produced binding energy profiles. Using experimentally verified TFBSs, sensitivity and specificity were calculated to determine the energy thresholds used to classify a given sequence as a binding site. The quality of prediction was estimated by prediction sensitivity, specificity and Receiver operating characteristic(ROC) analysis, as well as a *ψ*-test [25] comparing derived PWMs to characterized PWMs in databases.

## Materials and Methods

### Knowledge-based energy functions

In all of the following energy function tests, we assume rigid-body docking during the formation of protein/DNA complexes and neglect DNA deformation and conformational change of proteins induced by DNA binding. In other words, intra-protein and intra-DNA interactions are assumed to be unchanged during binding, as in our previous work [39]. The free energy of formation for a protein/DNA complex, Δ*G*, is approximated as:
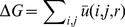
(1)where the summation is over all atomic pairs between atom types *i* and *j* that are a distance *r* apart. The details of how to derive this knowledge-based energy function (Supplementary Text S1), perform three new corrections(Supplementary Text S1) and estimate the corresponding results (Supplementary Table S1,S2) can be found in Supplementary Materials.

### Predicting transcription factor binding sites

**Figure 1:**
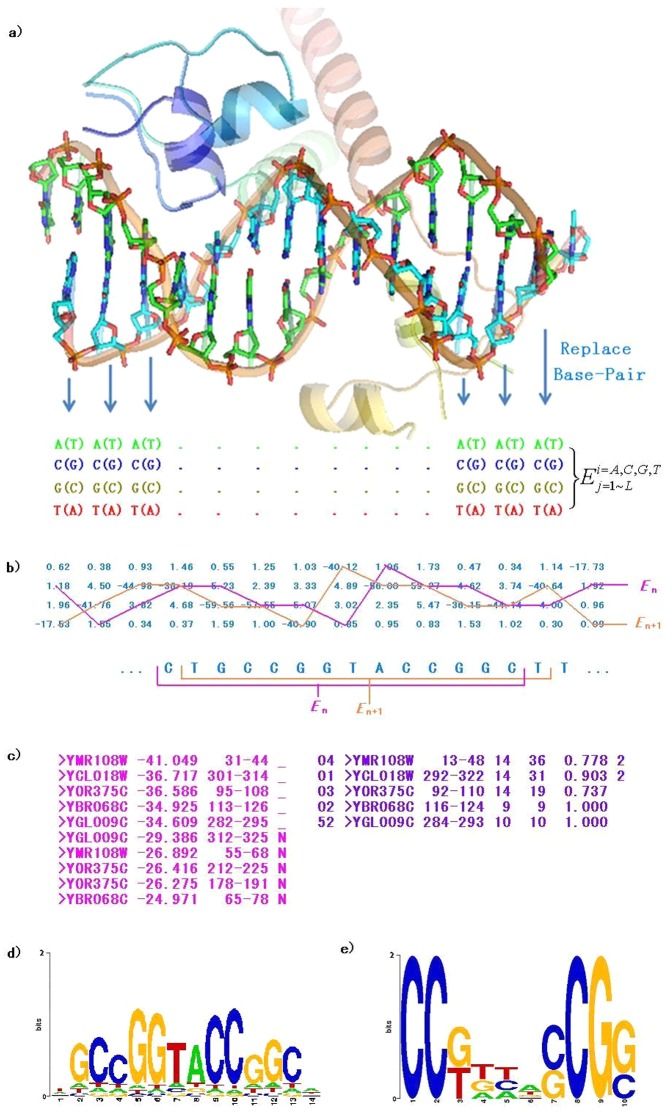
Structural based strategy for predicting transcription factor binding sites. An illustration of the structural based strategy for predicting transcription factor binding sites: a) A native structure of TF bound with TFBS (PDB id: 2ERE) is used as the structure template (image created by Pymol [54]). Each base pair in the TFBS with length L (length of TFBS is listed in Table 1) is replaced by four kinds of base pairs and only the energy of binding contributed by the substituted base pair and the TF is calculated after the replacement. A L×4 position energy matrix (PEM) is then generated for each TF. b) The sequences of ORFs (here for example YMR108W upstream −470∼−455) are threaded into a specific TF's position energy matrix to get the binding energy of a sequence with the TF. For example, the binding energy of a sequence CTGCCGGTACCGGC would be given as 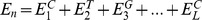, meanwhile the binding energy of sequence offset by a position, TGCCGGTACCGGCT would be given as 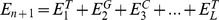. c) The binding energies of all sequences are sorted from lowest to highest (Left), and the binding sites from TRANSFAC and SCPD database (Right) are matched by overlapped position in the same ORF. Overlapped base pairs (Lo) with more than 50% of the binding sites in database (Ld) is considered as True Positive [55]. Note that some binding sites are much longer than that in native complex structure (Ln), in this case, we used Lo/Ln>50% as the criterion for classifying the site as the binding site. False Positive indicates a predicted TFBS not overlapping any TFBSs in the databases. True Negative indicates a TFBS in the databases overlapping with a predicted result not classified as a TFBS. d) Position energy matrix derived from PDB 2ere can be converted to PWM by Boltzmann formula [51]. The weblogo [56] of converted PWM where β = 0.05. Position 3∼12 is identical to part (e) position 1∼10. e) MA0324.1 NAME: LEU3 from the JASPAR CORE database [50] as comparison. We successful predicted most probability base pair on 9 out of 10 positions.

An illustration of our structural based strategy for predicting TFBSs is shown in Figure 1. Knowledge-based functions were evaluated for DNA sequence decoy discrimination, docking decoy discrimination, recovering native base pairs, and prediction of PWMs (refer to Supplementary Text S1,S2 and Table S1,S2 for details). The best performing knowledge-based function (Supplementary Table S1) was used to test the predictive power of the structural-based recognition of TFBSs. Structures of 16 *Saccharomyces cerevisiae* TFs were obtained from PDB and experimentally verified TFBS sites for these TFs were obtained from TRANSFAC [40] and the Promoter Database of *Saccharomyces cerevisiae* (SCPD) [41]. If two TFBSs for a given factor overlapped, the union of their overlapping regions was chosen (e.g. the union of TFBSs “chr1:1984–2007” and “chr1:1988–2012” is “chr1:1984–2012”). The sequence for promoter regions (500 bp upstream of TSS) of ORFs in *S. cerevisiae* strain S288C was downloaded from the Saccharomyces Genome Database (SGD) [42]. The experimentally characterized TFBSs were aligned to the promoters using Blat [43]. In total, 127 experimentally verified TFBSs were identified for the 16 TFs, distributed across the promoters of 87 Yeast ORFs. Table 1 includes a summary of the sites for these factors.

**Table 1:** Data Summary.

TF^a^	PDB chain id^b^	N_sites_ c	N_ORF_ d	N_residue_ e	L_TFBS_ f
GAL4	3coq_A,B	15	7	178	19
GCN4	1ysa_C,D	18	11	114	16
HAP1	1hwt_C,D,G,H	9	5	287	19
LEU3	2ere_A,B	5	5	120	14
MATA1	1yrn_A	1	1	49	13
MATALPHA2	1apl_C	10	7	59	12
MCM1	1mnm_A,B	26	21	166	20
MCM1_MATALPHA2	1mnm_A,B,C,D	1	1	320	25
NDT80	1mnn_A	1	1	290	13
PHO4	1a0a_A,B	4	1	126	16
PPR1	1pyi_A,B	1	1	158	14
PUT3	1zme_C,D	3	2	140	14
RAP1	1ign_A	24	18	189	18
TBP	1ytb_A	7	4	180	12
TFIIA	1ytf_C	1	1	192	13
TFIIA_TBP	1ytf_A,C	1	1	372	15

The transcription factors (TFs), their structure identifications and the number of experimentally verified transcription factor binding sites (TFBS) in yeast.

a Transcription factor name, ‘_’ denotes transcription factors found in complex.

b The structure used to represent the TF.

c The number of binding sites collected from TRANSFAC and SCPD.

d The number of ORFs these binding sites reside.

e The number of residues in the TF.

f The length of TFBS. Based on related PDB structure file, base-pairs that atoms are 10 Å away from the nearest atoms on the TF are excluded from the count.

PDB is the most extensive database of experimentally determined structures of proteins. We used the structure of a TF found in the native structure of the TF/DNA complex as the structure template when it was available in PDB. However, for some TFs, there are no known structures of the TF in complex with DNA. In these cases, if a structure exists with this TF in a complex with another TF, the substructures extracted from the TF/TF complexes were used instead. For example, there is no TF/DNA complex available in PDB for MCM1. However, there is structural information for the MCM1/MATAlpha2/DNA complex (PDB id:1mnm) and this was used to obtain the substructure of MCM1. The structural identifications of the 16 TFs used in this study can be found in Table 1.

The RaPvcFIRE energy function has been identified as the best performing knowledge-based energy function (Supplementary Table S1). The energy potential based on RaPvcFIRE was retrained on the same protein/DNA complex database after removal of 8 structures homologous with the 16 TFs (see Supplementary Table S3 for the list of all 208 structures used in the training set). The derived energy function was then used to predict the binding energy of TFs with TFBSs. For each of 16 TFs, we obtained the position-specific energy matrix (PEM) by substituting nucleotides at each position to each of the four possible nucleotide pairs (A-T, C-G, G-C, T-A). Binding energies of these base pairs at each position were calculated individually using the trained knowledge-based function. The PEM is a 4×*L* matrix (where *L* is the length of the TFBS found in the native TF/DNA complex structure) that can be converted to a PWM using the Boltzmann formula (34,56) with the pairwise additivity assumption. After obtaining the PEM for a specific TF, we scanned the sequence consisting of 500 bp upstream of the start codon of each ORF containing at least one known binding site with this PEM. The binding energies of 500-*L*+1 sequences in each promoter were sorted from low to high and the lowest 200 energies were kept. The energy cutoffs for classifying a sequence as a TFBS or not were determined by maximizing the prediction accuracy compared with experimentally collected TFBSs. The prediction accuracy was calculated as the sum of sensitivity [TP/(TP+FN)] and specificity [TP/(TP+FP)] (TP: true positive, FN: false negative, FP: false positive, definition as description in Figure 1c) as described previously [44]. A DNA sequence with *L* bases was classified as TFBS if its total binding energy is under the energy cutoff.

### Structural dependence

Recent research has shown that crystal structure quality greatly influences the sensitivity of TFBS prediction [27]. Unfortunately, high-quality TF/DNA complex structures exist for only a few factors. We therefore investigated methodologies for TFBS prediction when a crystal structure does not exist, but the prediction of TF/DNA structure is possible because DNA structure can be predicted along TF structure [45]. Random translocation and rotational perturbations were applied to the DNA in native complex structures, leading to newly generated structures that differ from the original complex structures by as much as 4 Å root mean square deviation (RMSD). These newly generated structures were grouped according to their RMSD values, with RMSD between (n−1,n] grouped as RMSD∼n (n = 1,2,3,4). For example, group RMSD∼1 contains 16 perturbed structures that differ from their native structures by RMSDs ranging from (0,1]. Predictions were made separately based on these newly formed structures as structure template. The RMSD values of these perturbed structure templates can be found in the Supplementary Table S4. In total, we obtained 80 structures including the original native structures for 16 different TFs, and 4 perturbed structures for each TF.

### Methodologies and Tests

**Figure 2:**
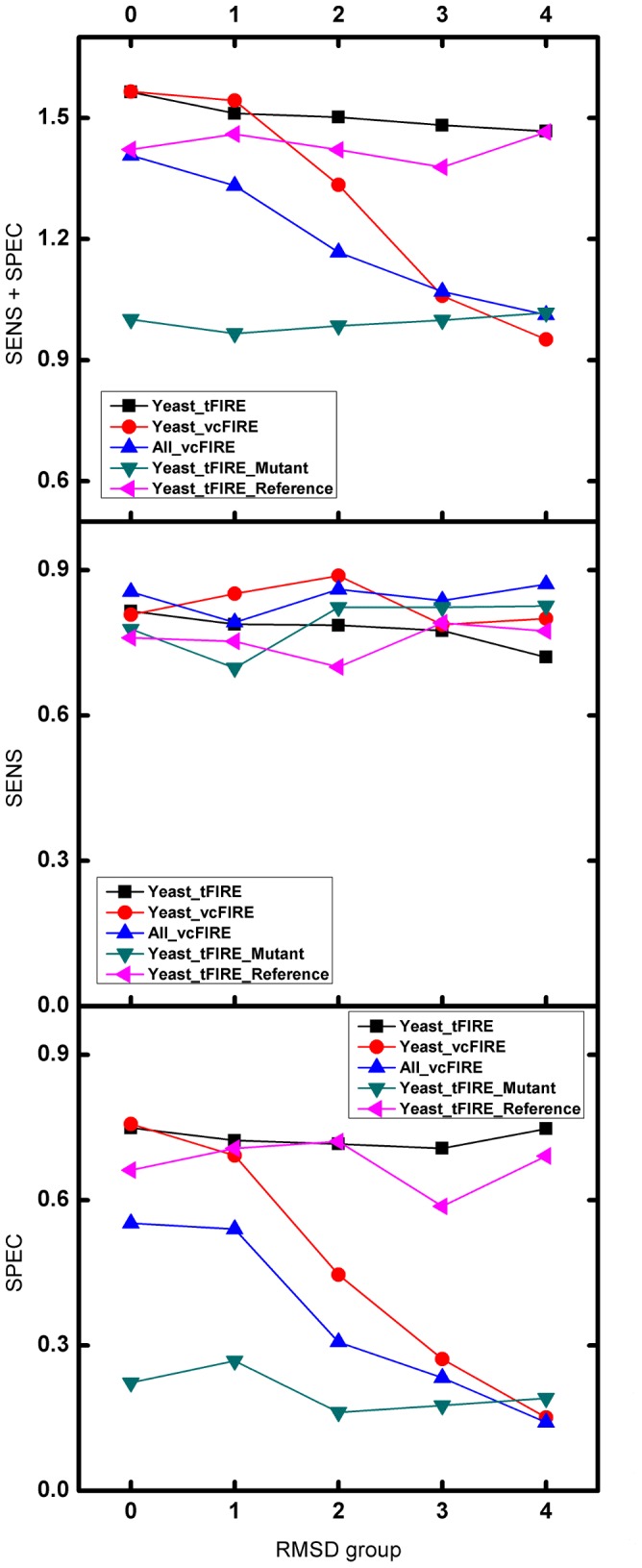
Prediction accuracy on different structures set. Prediction accuracy on different structures set, where RMSD∼0 denotes using native structure in PDB. Group RMSD∼n(n = 1,2,3,4) indicate that structure templates have the same protein structure with native structure, but the DNA have been changed by basic translocation and rotation. The RMSD between the changed DNA and the native DNA part is (n-1,n]. Different training sets are shown in various symbols. a) Each prediction in Yeast_tFIRE is using the structure template itself as the training set. b) All_vcFIRE using all 212 TF/DNA structures in PDB which we have tested our energy functions on in a previous study [39]. c) Yeast_vcFIRE set using all yeast TF/DNA structures as the training set, including the 16 structures shown in Table 1. d) Yeast_tFIRE_Mutant set training each energy function with one structure, but each position of the DNA sequence is mutated to a random base pair with equal possibility. e) Yeast_tFIRE_Reference set all non-lowest energy values from Yeast_tFIRE to zero.

Our tests found that methods used to train an energy function with structures selected from PDB (e.g. vcFIRE) are overly sensitive to the relative position of the TF to a binding site. This can lead to difficulties in employing computationally predicted 3D structures in structural-based TFBS predictions and limits the effectiveness of our approach. We present here a strategy to overcome the over sensitivity of traditional methods. This strategy can briefly be described as training the energy function by the structure template itself (tFIRE). Other tests related to this strategy were also performed. As shown in Figure 2, a total of five parallel prediction sets were constructed as described below.

#### Prediction Set 1 (Yeast_tFIRE)

This set denotes our new proposed strategy. The structure template itself is used in the training set (for 16 factors with 5 RMSD groups, a total of 80 energy functions are trained and tested on the structure template).

#### Prediction Set 2 (All_vcFIRE)

This set denotes our TFBS predictions employing the energy function described in our previous work [39]. It consists of all 212 TF/DNA structures in PDB which we have tested/trained using energy functions. The maximum sequence identity between each two structures in this set is below 35% as culled by PISCES [46]. To evaluate the general applicability of this method, structures of proteins with sequences identical to the 16 yeast structures described above were also removed from this training set (a protein was considered identical if the BLAT score was more than 2500). Here only one energy function is trained, predictions are made over 80 structure templates.

#### Prediction Set 3 (Yeast_vcFIRE)

We also investigated whether training the energy function using only structures characterized from yeast produced better results. In this set, all native TF/DNA structures from yeast were used in the training set, including the 16 structures shown in Table 1. Similar to the All_vcFIRE set, only one energy function is trained but predictions are made over 80 structure templates.

#### Prediction Set 4 (Yeast_tFIRE_Mutant)

To estimate the contribution of DNA sequence in tFIRE, each position of the DNA sequence was mutated to a random base pair with equal possibility prior to training to form 64 decoys as structure templates. With 16 native structures, a total of 80 energy functions were trained and tested on the structure template itself over 5 RMSD groups. The mutant DNA sequences can be found in Supplementary Table S5.

#### Prediction Set 5 (Yeast_tFIRE_Reference)

In each of the tests described above, the energies of the high-information content positions in the TFBS are much lower than the energies in the low-information content positions. For example, in Figure 1b, the energy for the T-A base pair in the first position is −17.53 while the other possible base pairs have energies greater than zero. In order to investigate if the information content of motif was affecting the success of the prediction, we set the energies of all non-lowest energy base pairs to zero and examined the prediction accuracy.

## Results

### Comparison to alternative approaches

If a given factor has a well-characterized PWM, it is straightforward to identify potential TFBSs. However, if a well-characterized PWM does not exist, there are several strategies that can be followed. Furthermore, if a commonly regulated set of sequences thought to contain a common motif exists, *de novo* motif discovery approaches can be used to identify statistically overrepresented motifs in these sequences. The PWMs corresponding to these motifs can then be used to scan through the sequences to find potential TFBSs. To compare the use of 3D structure information in the prediction of potential TFBSs with these more traditional approaches, we list in Table 2, a comparison of our structure-based approach with several of the most popular *de novo* motif discovery methods (MEME [47], AlignACE [48] and BioProspector [49]). For most TFs, employing the 3D structure can produce better prediction accuracy. Motif discovery methods suffer when working with such small sequence sets. Furthermore, if no experimental data exists to determine sequences with potential common regulatory motifs exists, but a 3D complex structure of the TF/DNA complex is available, the PWM can be generated using the methods described here. There are also cases for which *de novo* motif discovery methods perform better than 3D structure methods. The use of AlignACE, BioProspector for GAL4 as well as the use of MEME for HAP1 outperform our structure-based approach. We speculate that this is because the promoter regions of the commonly regulated ORFs have a strong enrichment in these binding sites.

**Table 2:** Comparison to other methods.

Transcription Factor	N_S_ a	N_O_ b	Our			AlignACE^d^			BioProspector^e^			MEME^f^		
			SE^c^	SP^c^	AUC^c^	SE^c^	SP^c^	AUC^c^	SE^c^	SP^c^	AUC^c^	SE^c^	SP^c^	AUC^c^
MCM1_MATALPHA2	1	1	1.00	1.00	1.00	-	-	-	-	-	-	-	-	-
PPR1	1	1	1.00	1.00	1.00	-	-	-	-	-	-	-	-	-
NDT80	1	1	1.00	1.00	1.00	-	-	-	-	-	-	-	-	-
LEU3	5	5	1.00	1.00	1.00	0.40	1.00	0.76	1.00	0.83	0.98	0.60	1.00	0.88
MATA1	1	1	1.00	1.00	1.00	1.00	0.07	0.42	-	-	-	-	-	-
TFIIA_TBP	1	1	1.00	0.50	0.91	-	-	-	-	-	-	-	-	-
GAL4	15	7	0.80	1.00	0.90	1.00	1.00	1.00	0.60	1.00	0.91	0.67	0.62	0.80
MCM1	26	21	0.96	0.49	0.86	0.65	0.85	0.85	0.65	0.94	0.83	0.85	0.16	0.61
MATALPHA2	10	7	0.50	0.83	0.83	0.90	0.08	0.47	0.30	1.00	0.68	0.10	1.00	0.64
TBP	7	4	0.71	0.83	0.81	1.00	0.07	0.57	1.00	0.12	0.60	0.86	0.06	0.39
PHO4	4	1	1.00	0.29	0.78	1.00	0.44	0.78	-	-	-	-	-	-
PUT3	3	2	0.67	1.00	0.76	1.00	0.17	0.59	1.00	0.05	0.46	0.33	0.50	0.67
GCN4	18	11	0.39	0.78	0.72	0.56	0.06	0.29	0.17	1.00	0.63	0.06	1.00	0.44
HAP1	9	5	0.89	0.12	0.68	0.75	0.06	0.56	0.89	0.06	0.46	1.00	0.20	0.73
RAP1	24	18	0.12	1.00	0.66	0.11	1.00	0.20	0.41	0.07	0.22	0.83	0.14	0.58
TFIIA	1	1	1.00	0.14	0.62	-	-	-	-	-	-	-	-	-
Average^g^	7.94	5.44	0.67	0.78	0.80	0.71	0.48	0.59	0.67	0.56	0.64	0.59	0.52	0.64

Most of tFIRE prediction is better than others. Except the use of AlignACE, BioProspector for GAL4 as well as the use of MEME for HAP1 outperform our structure-based approach.

a N_S_: Number of sites collected from TRANSFAC and SCPD.

b N_O_: Number of ORFs these binding sites taking place.

c SE: Sensitivity [TP/(TP+FN)]. SP: Specificity [TP/(TP+FP)]. AUC: Area Under Receiver operating characteristic Curve.

d AlignACE [48] v4.0 result using parameter as: number of columns = L_TFBS_.

e BioProspector [49] result as: motif width = L_TFBS_; top motifs to report = N_Sites_.

f MEME [47] v4.3.0 result as: maximum motif width = L_TFBS_; maximum sites = N_Sites_; maximum motif number = N_Sites_.

g Average value over LEU3, GAL4, MCM1, MATALPHA2, TBP, PUT3, GCN4, HAP1, RAP1.

### Comparison of structure-based strategies

**Figure 3:**
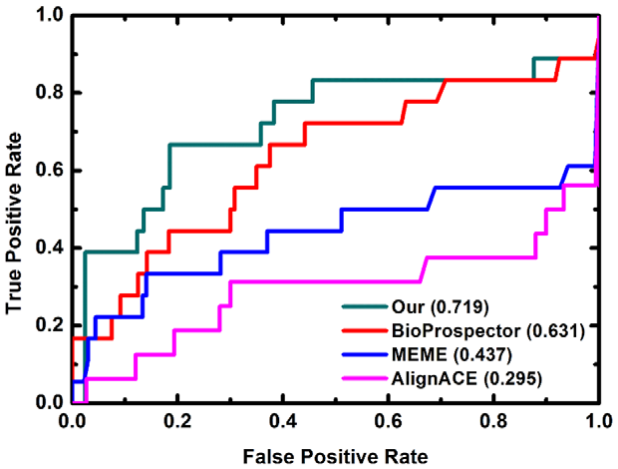
Performance of three *de novo* methods and tFIRE using GCN4 as an example. The prediction results of binding are shown in the ROC plot. The ROC curves were generated by plotting the true positive rate [TP/(TP+FN)] (y-axis) against the false positive rate [FP/(TN+FP)] (x-axis). The AUC values for the three methods is shown in parentheses.

We tested a number of different training sets, as shown in Figure 3. A summary of each of the various training sets is described below. First, training the energy function and making predictions using the native structure itself gives the best prediction (see results for Yeast_tFIRE with group RMSD∼0). The sensitivity is 0.82, which is slightly lower than the 0.85 sensitivity of the All_vcFIRE set group RMSD∼0. However, the specificity is 0.75, higher than 0.55 of the All_vcFIRE set group RMSD∼0. This indicates a 12% improvement on accuracy (sensitivity plus specificity). Furthermore, the prediction accuracy of the Yeast_tFIRE set is very robust to the structure template we are using. The average accuracy along 5 RMSD group is 1.51+/−0.04. Meanwhile, the All_vcFIRE set can only achieve an accuracy of 1.20+/−0.17.

Second, the prediction accuracy of All_vcFIRE decreases if the DNA component is different from its native structure. This confirms the structural dependence along the relative position between TF and DNA, similar to what has been reported by Alamanova and colleagues [27]. This structural dependence is also seen with the Yeast_vcFIRE group. Among these training sets, training with structures of factors only characterized in yeast (i.e. Yeast_vcFIRE set) produced better results than with all non-homology TF/DNA complex structures (i.e. All_vcFIRE set). We also note that for these two sets, the results of site prediction using the training set with RMSD∼4 indicates that the All_vcFIRE set is superior. We speculate that this is caused by X-Ray resolution error in that all of the structures used have resolution below 3 Å (except 1pyi with 3.2 Å resolution).

Third, to further assess the importance of the sequence in the complex structure, we tested the training group Yeast_tFIRE_Mutant, which replaces the DNA sequence from native structure with random base pairs. This group performed worst, with an average accuracy along 5 RMSD groups of 1.00+/−0.02. This emphasizes the importance of the TFBS sequence in the structure template for TFBS prediction.

In summary, we observed that the prediction accuracy of Yeast_tFIRE_Reference was reduced by about 5% along 5 RMSD groups with an average accuracy of 1.43+/−0.04. This indicates that the degenerate positions are useful (for example, the binding of some factors may be controlled by the combination of two or more degenerate positions). The full results for these prediction sets can be found in Supplementary Tables S6 and S7.

A summary of the prediction accuracy for the various methods is shown in Table 3. For MCM1, the prediction specificity is only 0.49, but 22 of total 26 predicted sites are ranked as the top scoring windows in the corresponding promoter. It is possible that there are viable binding sites in these sequences that have not been experimentally confirmed, artificially lowering the specificity.

**Table 3:** Prediction accuracy.

Transcription Factor^a^	TP^b^	FN^b^	FP^b^	SE^b^	SP^b^	SE+SP^b^	AUC^c^	N_TSites_ d	N_Sites_ e	N_TORF_ f	N_ORF_ g
MCM1_MATALPHA2	1	0	0	1.00	1.00	2.00	1.00	1	1	1	1
PPR1	1	0	0	1.00	1.00	2.00	1.00	1	1	1	1
NDT80	1	0	0	1.00	1.00	2.00	1.00	1	1	1	1
LEU3	5	0	0	1.00	1.00	2.00	1.00	5	5	5	5
MATA1	1	0	0	1.00	1.00	2.00	1.00	1	1	1	1
TFIIA_TBP	1	0	1	1.00	0.50	1.50	0.91	0	1	0	1
GAL4	12	3	0	0.80	1.00	1.80	0.90	12	15	7	7
MCM1	25	1	26	0.96	0.49	1.45	0.86	22	26	18	21
MATALPHA2	5	5	1	0.50	0.83	1.33	0.83	6	10	5	7
TBP	5	2	1	0.71	0.83	1.55	0.81	4	7	4	4
PHO4	4	0	10	1.00	0.29	1.29	0.78	1	4	1	1
PUT3	2	1	0	0.67	1.00	1.67	0.76	2	3	2	2
GCN4	7	11	2	0.39	0.78	1.17	0.72	8	18	5	11
HAP1	8	1	56	0.89	0.12	1.01	0.68	3	9	2	5
RAP1	3	21	0	0.12	1.00	1.12	0.66	11	24	8	18
TFIIA	1	0	6	1.00	0.14	1.14	0.63	0	1	0	1
Average	5.13	2.81	6.44	0.81	0.75	1.56	0.85	4.88	7.94	3.81	5.44
Standard Deviation	6.17	5.64	14.81	0.27	0.33	0.37	0.13	5.93	8.48	4.53	6.26

tFIRE can achieve a average AUC at 0.85±0.13 and many of the predictions are top ranked TFBS.

a Transcription factor name, ‘_’ denotes complex by two transcription factors.

b TP: true positive. FN: false negative. FP: false positive. SE: Sensitivity [TP/(TP+FN)]. SP: Specificity [TP/(TP+FP)].

c Area Under Receiver operating characteristic Curve.

d N_TSites_: Number of sites ranked top, the higher the better discrimination ability in ORF.

e N_Sites_: Number of sites collected from TRANSFAC and SCPD.

f N_TORF_: Number of prediction on how many ORFs achieved top ranked TFBS.

g N_ORF_: Number of ORFs these binding sites taking place.

### Comparison to use of PWM alone

There are 12 TFs from our test set with PWMs in JASPAR [50]. We selected these PWMs to form a JAS-PWM set (there is no PWM for PPR1, TFIIA, MCM1_MATALPHA2, or TFIIA_TBP in JASPAR).

For prediction, a PEM can be converted to a PWM (Pre-PWM) with the Boltzmann formula [51]:
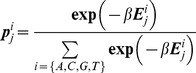
(2)where 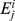 is the position energy at position *j* for base pair *i*,*β* = 0.05.

We also define an Even-PWM as where the expected frequency of A,C,G,T on each position is 0.25 as for the reference.

As shown in Table 4, the result of Pre-PWM is better than Even-PWM (paired *t*-test two-tailed p-value equals 0.0005; p-values were calculated using paired *t*-test from CPAN Perl module Statistics::Distributions and Statistics::DependantTTest).

**Table 4:** PWM similarity to well-characterized PWM.

Transcription Factor	Pre-PWM^b^	Even-PWM^c^
GAL4	0.15	0.32
GCN4	0.12	0.32
HAP1	0.33	0.40
LEU3	0.22	0.48
MATA10	0.73	0.70
MATALPHA20	0.31	0.64
MCM10	0.34	0.41
NDT80	0.11	0.44
PHO4	0.19	0.54
PUT3	0.40	0.52
RAP1	0.49	0.52
TBP0	0.17	0.43
Average	0.30	0.48
Standard Deviation	0.18	0.12

*ψ*-test of predicted PWM to experimental PWM demonstrates prediction accuracy. The smaller *ψ*-test compared to Even-PWM the better.

This table shows the ψ-test [25] value of each TF's predicted PWM(Pre-PWM) via experimental PWM collected from JASPAR(33) while Pre-PWMs are converted from PEM by Boltzmann formula [51].

Also ψ-test of Even-PWM with an equal frequency of 0.25 for A,C,G,T at each position compared to experimental PWM.

### PWM for TF-TF complex

**Figure 4:**
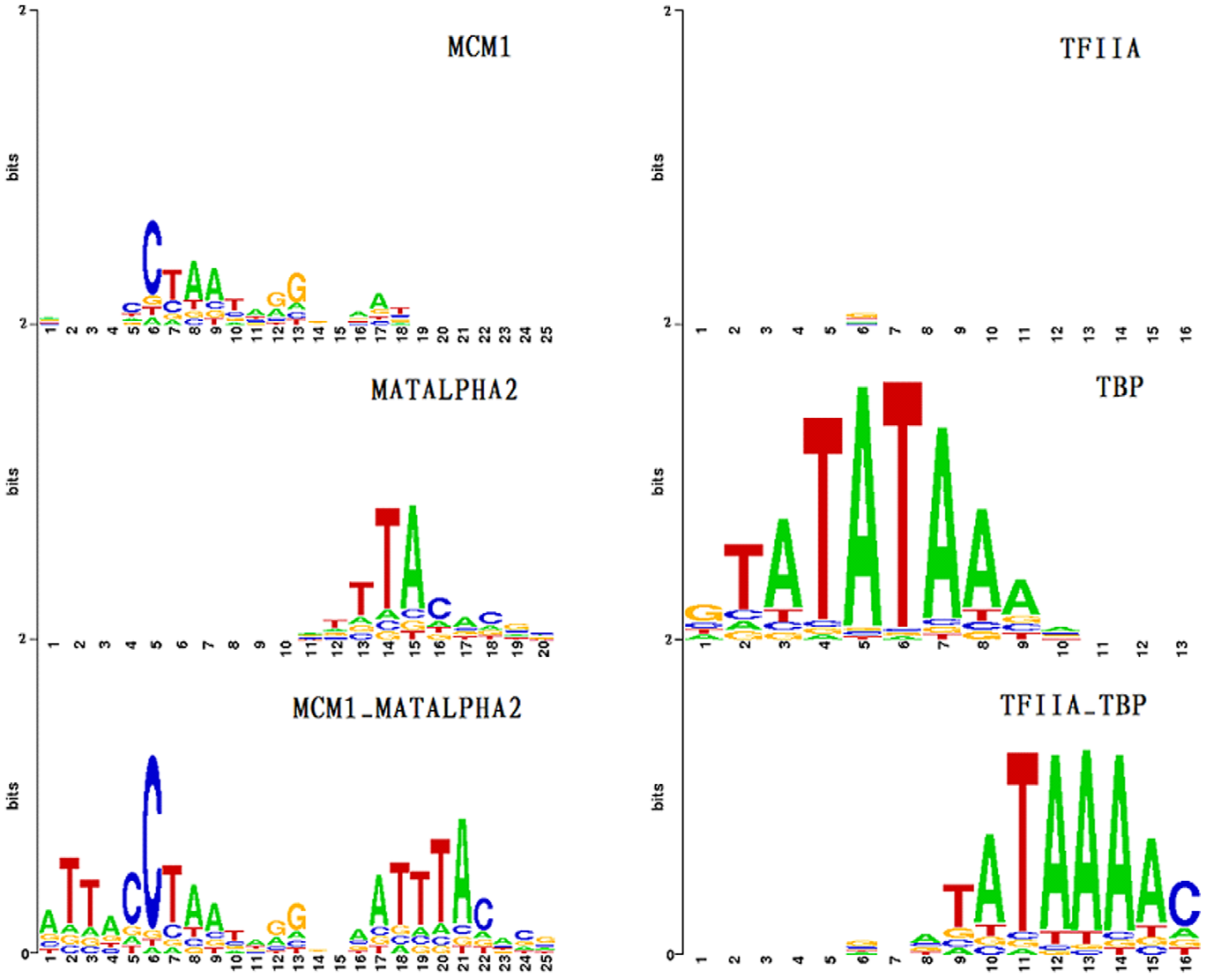
Predicted PWMs of two TF-TF complexes and their subunits. The PWM of MCM1/MATALPHA2 complex is most likely to be a superposition of the MCM1 and MATALPHA2 PWMs. This indicates that MCM1 and MATALPHA2 both have strong binding affinity to DNA. Their complex's binding pattern contains both MCM1 and MATALPHA2 binding patterns. Conversely, TFIIA, which does not bind DNA directly, shows a very weak PWM, as expected. The TFIIA/TBP complex leads to a very different PWM prediction as compared to TBP alone. This indicates that TFIIA may not bind DNA directly, but it may alter the TBP structure [52].

Many transcription factors do not bind alone on the genome but instead bind as part of complexes, often forming TF-TF dimers. For example, MCM1 binds in a complex with MATALPHA2 and TFIIA binds together with TBP. We generated the predicted PWMs of MCM1 and MATALPHA2 as the MCM1/MATALPHA2 complex (Figure 4). The PWM of MCM1/MATALPHA2 complex is most likely to be a superposition of MCM1 and MATALPHA2 PWMs. This indicates that both MCM1 and MATALPHA2 have strong binding infinity to DNA, so that their complex's binding pattern contains both MCM1 and MATALPHA2 binding patterns.

TFIIA, which does not bind DNA directly, shows a very weak PWM, as expected. The TFIIA/TBP complex leads to a very different PWM prediction as compared to TBP alone. This is due to the fact that the DNA sequence used to train the energy function in the TBP-DNA complex is GTAT**A**TAAAACGG, while the sequence in the TFIIA_TBP-DNA complex is TGTAT**G**TATAAAAC. This indicates that TFIIA may not contribute to transcription by binding DNA directly, but may be involved by altering the TBP structure [52].

## Discussion

We propose three new corrections based on volume fraction corrected, pseudo-count added, ideal-gas reference (vcFIRE) state for protein/DNA interactions. They are 1) reweighting of observed atom pairs, 2) new smoothing approaches and 3) a dipolar approximation. The new proposed methods further extend the statistical energy function derived with the distance-scaled FIRE (DFIRE) state that was originally developed for proteins [38], [53] and applied to protein/DNA interactions (DDNA [36], DDNA2 [39], the energy function obtained by training all non-homology structures in PDB by vcFIRE). Improvements over DDNA2 by FIRE-based energy functions are observed in four different tests: threading and docking decoy discriminations, recovery of native base pairs, and prediction of binding profiles. The improvements are not only reproduced by each correction but also by the combinations of these corrections. These corrections are useful for developing new knowledge-based energy functions, and for improving prediction methods based on new energy functions.

We have shown here that combining knowledge-based energy functions (e.g. with structural data) and experimental binding site data into TFBS predictions leads to improved methods to identify potential TFBSs. Our results also confirm the importance of TFBS sequence of structure template in recognizing TFs. The Yeast_tFIRE_Reference set also shows that the recognition of the lowest energy base pair is not the most critical component of TFBS prediction. Training a knowledge-based energy function with a single TF/DNA complex is conceptually similar to scanning for TFBSs with a consensus sequence. However, if a 3D structure of the TF/DNA complex exists, the tFIRE approach can be used even if there are too few confirmed binding sites to construct a consensus sequence.

One limitation of tFIRE is the availability of TF/DNA complex structures, but the results for the Yeast_tFIRE set shows that the experimentally solved TF/DNA complex structure may not be a prerequisite to predict TFBSs. This new proposed strategy is insensitive to the related position between DNA and TF. Along with a protein/DNA docking approach, this strategy could be widely applied to not only DNA-binding proteins but also to other regulatory proteins. Furthermore, homology modeling can be used to build the protein structure if there is no available structure in databases. By this approach, we can potentially predict binding sites for all regulatory proteins, which is a critical step in constructing of gene expression regulatory networks.

## Supporting Information

Table S1 **Energy function estimation result.** Bold indicates the best of these eight methods. ^a^Method denotes energy functions derived with different approaches, as explained in “Systems and Methods” section. ‘R’ denotes the use of reweight of observed atom pairs. ‘a’ denotes the use of smaller bins with smoothing. ‘P’ denotes thhe use of dipolar approximation. ^b^Threading decoys of 51 complexes collected by Kono and Sarai [1], the ratio how many structures out of 50,000 with random DNA sequences have higher energy than the native structure. ^c^ *Z-Score* measures the ability of an energy function to discriminate a native DNA sequence from randomly generated DNA sequences, the lower the better. ^d^Near-native docking decoy sets of 45 protein-DNA complexes from Robertson and Varani [2], the ratio how many structures out of 2000 lowest-RMSD decoys have higher energy than the native structure. Decoys for each complex generated from restraints around native complex structures by FTDock. ^e^ *Z-Score* measures the ability of an energy function to discriminate a native DNA sequence from its near-native docking decoys. ^f^The median value of the lowest rmsd structure in top five decoys ranked by various energy functions. The best possible median value is 0.44 Å. ^g^Base-pair recovery rates average on ten-fold cross validation. Randomly selected 200 complexes are divided randomly into 10 parts (“folds”). In ten tests, nine folds are used for training and the remaining fold is for testing. ^h^Accuracy of PWM prediction based on *ψ*-test values for 19 complexes by various methods.
(DOC)

Table S2 **The rmsd value of the lowest energy complex structure selected by various energy functions.** This tables using 2000 lowest-RMSD docking decoys as described before, shows the lowest energy structure's RMSD to the native structures. ^a^Protein data bank identification code. ^b^The degree of overall DNA deformation. ^c^The lowest RMSD decoy. ^d^The median value of the lowest rmsd structure in top five decoys ranked by various energy functions. ^e^How many decoy set successful discriminated the lowest RMSD structures.
(DOC)

Table S3 **PDB list for predict TFBS.**
(DOC)

Table S4 **RMSD list for Different Structure Templates.**
(DOC)

Table S5 **Training set for Yeast_Self_Mutant.** N_mutated_ bases have been mutated from N_sequence_ in Yeast_Self_Mutant set.
(DOC)

Table S6 **Prediction Results.**
(DOC)

Table S7 **Prediction Result for Top Ranked ORFs.** To suppress the effect of experimental-unknown binding sites and the potential difference between chromosomes, the top ranked ORF numbers were shown in here. These number denotes in each set, for how many ORFs, our lowest energy sequences are overlapping the experimental binding sites. 1. Kono H, Sarai A (1999) Structure-based prediction of DNA target sites by regulatory proteins. Proteins-Structure Function and Genetics 35: 114–131. 2. Robertson TA, Varani G (2007) An all-atom, distance-dependent scoring function for the prediction of protein-DNA interactions from structure. Proteins-Structure Function and Bioinformatics 66: 359–374.
(DOC)

Text S1 **Derivation of Knowledge-based Energy functions.**
(DOC)

Text S2 **PBD structures used in decoy tests.**
(DOC)
