# Correction: A Structural-Based Strategy for Recognition of Transcription Factor Binding Sites

**DOI:** 10.1371/annotation/0312e1be-336d-430a-aa90-101bbb44f8db

**Published:** 2013-10-15

**Authors:** Beisi Xu, Dustin E. Schones, Yongmei Wang, Haojun Liang, Guohui Li

There were errors in the Funding section. The correct funding information is as follows:

This work was supported by grants from the National Natural Science Foundation of China (31070641), the National Basic Research Program of China (2012CB721002), the “Hundreds Talents Program” of the Chinese Academy of Sciences, the National High Technology Research and Development Program of China (863 project) (No. 2012AA01A305), and the National Science Foundation of China under Contract No. 91227126. The funders had no role in the study design, data collection and analysis, decision to publish or preparation of the manuscript. 

