# Supplementary material for: A Structural-Based Strategy for Recognition of Transcription Factor Binding Sites
Source: PLoS One. 2013 Jan 8;8(1):e52460. doi: 10.1371/journal.pone.0052460 (PMC3540023; doi:10.1371/journal.pone.0052460)
Supplement: Table S1 — Energy function estimation result. Bold indicates the best of these eight methods. aMethod denotes energy functions derived with different approaches, as explained in “Systems and Methods” section. ‘R’ denotes the use of reweight of observed atom pairs. ‘a’ denotes the use of smaller bins with smoothing. ‘P’ denotes thhe use of dipolar approximation. bThreading decoys of 51 complexes collected by Kono and Sarai [1], the ratio how many structures out of 50,000 with random DNA sequences have higher energy than the native structure. c Z-Score measures the ability of an energy function to discriminate a native DNA sequence from randomly generated DNA sequences, the lower the better. dNear-native docking decoy sets of 45 protein-DNA complexes from Robertson and Varani [2], the ratio how many structures out of 2000 lowest-RMSD decoys have higher energy than the native structure. Decoys for each complex generated from restraints around native complex structures by FTDock. e Z-Score measures the ability of an energy function to discriminate a native DNA sequence from its near-native docking decoys. fThe median value of the lowest rmsd structure in top five decoys ranked by various energy functions. The best possible median value is 0.44 Å. gBase-pair recovery rates average on ten-fold cross validation. Randomly selected 200 complexes are divided randomly into 10 parts (“folds”). In ten tests, nine folds are used for training and the remaining fold is for testing. hAccuracy of PWM prediction based on ψ-test values for 19 complexes by various methods. (DOC) [file pone.0052460.s001.doc]

**Table S1. Energy function estimation result**

Bold indicates the best of these eight methods.

| Method*^a^* | Thread Suc%^b^ | Thread Z-Score^c^ | Dock Suc%^d^ | Dock Z-Score^e^ | Median^f^ | Recover%^g^ | PWM^h^ |
| --- | --- | --- | --- | --- | --- | --- | --- |
| RaPvcFIRE | 97.2 | **-3.28** | **100.0** | **-4.08** | **0.53** | **44.9** | **0.302** |
| RPvcFIRE | **97.7** | -3.27 | **100.0** | -3.91 | 0.54 | 44.1 | 0.309 |
| aPvcFIRE | 96.5 | -3.18 | **100.0** | -3.59 | 0.54 | 44.5 | 0.307 |
| PvcFIRE | 97.0 | -3.12 | 99.2 | -3.04 | 0.55 | 43.4 | 0.315 |
| RavcFIRE | 96.7 | -3.05 | 99.9 | -2.83 | 0.54 | 42.8 | 0.321 |
| RvcFIRE | 96.3 | -2.93 | 99.9 | -2.78 | 0.56 | 42.1 | 0.328 |
| avcFIRE | 97.1 | -2.97 | 99.9 | -2.91 | **0.53** | 41.4 | 0.325 |
| vcFIRE | 96.6 | -2.86 | 99.9 | -2.86 | 0.54 | 40.4 | 0.332 |

1. Method denotes energy functions derived with different approaches, as explained in “Systems and Methods” section. ‘R’ denotes the use of reweight of observed atom pairs. ‘a’ denotes the use of smaller bins with smoothing. ‘P’ denotes thhe use of dipolar approximation.
2. Threading decoys of 51 complexes collected by Kono and Sarai, the ratio how many structures out of 50,000 with random DNA sequences have higher energy than the native structure.
3. *Z-Score* measures the ability of an energy function to discriminate a native DNA sequence from randomly generated DNA sequences, the lower the better.
4. Near-native docking decoy sets of 45 protein-DNA complexes from Robertson and Varani, the ratio how many structures out of 2000 lowest-RMSD decoys have higher energy than the native structure. Decoys for each complex generated from restraints around native complex structures by FTDock.
5. *Z-Score* measures the ability of an energy function to discriminate a native DNA sequence from its near-native docking decoys.
6. The median value of the lowest rmsd structure in top five decoys ranked by various energy functions. The best possible median value is 0.44Å.
7. Base-pair recovery rates average on ten-fold cross validation. Randomly selected 200 complexes are divided randomly into 10 parts (“folds”). In ten tests, nine folds are used for training and the remaining fold is for testing.
8. Accuracy of PWM prediction based on ψ-test values for 19 complexes by various methods.

1. Kono H, Sarai A (1999) Structure-based prediction of DNA target sites by regulatory proteins. Proteins-Structure Function and Genetics 35: 114-131.

2. Robertson TA, Varani G (2007) An all-atom, distance-dependent scoring function for the prediction of protein-DNA interactions from structure. Proteins-Structure Function and Bioinformatics 66: 359-374.
