# Supplementary material for: A Structural-Based Strategy for Recognition of Transcription Factor Binding Sites
Source: PLoS One. 2013 Jan 8;8(1):e52460. doi: 10.1371/journal.pone.0052460 (PMC3540023; doi:10.1371/journal.pone.0052460)
Supplement: Table S2 — The rmsd value of the lowest energy complex structure selected by various energy functions. This tables using 2000 lowest-RMSD docking decoys as described before, shows the lowest energy structure's RMSD to the native structures. aProtein data bank identification code. bThe degree of overall DNA deformation. cThe lowest RMSD decoy. dThe median value of the lowest rmsd structure in top five decoys ranked by various energy functions. eHow many decoy set successful discriminated the lowest RMSD structures. (DOC) [file pone.0052460.s002.doc]

**S4. The rmsd value of the lowest energy complex structure selected by various energy functions**

| PDB ID^a^ | **Γ(°)^b^** | vcFIRE | avcFIRE | RvcFIRE | RavcFIRE | PvcFIRE | aPvcFIRE | RPvcFIRE | RaPvcFIRE | Lowest^c^ |
| --- | --- | --- | --- | --- | --- | --- | --- | --- | --- | --- |
| 1a1i | 6.21 | 0.23 | 0.23 | 0.23 | 0.23 | **0.17** | 0.23 | 0.23 | 0.23 | 0.17 |
| 1a3q | 5.08 | 0.88 | 0.87 | 0.88 | 0.88 | **0.42** | **0.42** | 1.05 | **0.42** | 0.42 |
| 1a73 | 6.56 | **0.45** | **0.45** | **0.45** | **0.45** | **0.45** | **0.45** | **0.45** | **0.45** | 0.45 |
| 1au7 | 8.48 | **0.50** | **0.50** | **0.50** | **0.50** | **0.50** | 0.53 | **0.50** | 0.53 | 0.50 |
| 1b3t | 7.74 | 0.90 | 0.90 | 0.90 | 0.88 | 0.67 | 0.62 | 0.65 | 0.62 | 0.51 |
| 1bc8 | 6.10 | 0.87 | 0.87 | 0.87 | 0.87 | 0.87 | **0.33** | 0.87 | 0.87 | 0.33 |
| 1bdt | 6.41 | 0.54 | 0.54 | 0.54 | 0.54 | 0.54 | 0.54 | 0.54 | 0.54 | 0.46 |
| 1bl0 | 5.88 | 0.40 | 0.37 | 0.94 | 0.94 | 0.37 | 0.37 | 0.37 | 2.56 | 0.35 |
| 1ckq | 12.29 | 0.61 | 0.61 | 0.41 | 0.41 | 23.60 | 0.61 | 0.61 | 0.61 | 0.39 |
| 1d02 | 13.62 | **0.43** | **0.43** | **0.43** | **0.43** | 0.70 | **0.43** | **0.43** | **0.43** | 0.43 |
| 1dfm | 5.05 | **0.40** | **0.40** | 1.00 | 1.24 | 29.88 | **0.40** | **0.40** | **0.40** | 0.40 |
| 1dmu | 9.12 | **0.25** | **0.25** | **0.25** | **0.25** | **0.25** | **0.25** | **0.25** | **0.25** | 0.25 |
| 1dsz | 4.38 | 0.40 | 0.40 | 0.40 | 0.40 | 0.40 | 0.40 | 0.40 | **0.24** | 0.24 |
| 1eon | 13.41 | 0.61 | **0.42** | 0.61 | **0.42** | 23.11 | 28.95 | 0.61 | 0.61 | 0.42 |
| 1f4k | 4.80 | 0.50 | 0.53 | 0.53 | 0.53 | 0.50 | 0.45 | 0.50 | 0.53 | 0.44 |
| 1fjl | 4.95 | 0.51 | 0.51 | 0.51 | 0.51 | 0.73 | 0.73 | 0.51 | 0.73 | 0.46 |
| 1g9z | 7.17 | 0.49 | 0.49 | 0.49 | 0.49 | **0.41** | **0.41** | **0.41** | **0.41** | 0.41 |
| 1h8a_a | 4.82 | 0.70 | 1.30 | 0.80 | 0.56 | 0.75 | 0.75 | 0.68 | 0.75 | 0.53 |
| 1h8a_b | 4.82 | **0.11** | **0.11** | **0.11** | **0.11** | **0.11** | **0.11** | **0.11** | **0.11** | 0.11 |
| 1hlv | 4.53 | **0.34** | 0.48 | **0.34** | 0.48 | 0.48 | 0.48 | 0.48 | 0.48 | 0.34 |
| 1hwt | 4.13 | 1.64 | 1.35 | 1.12 | 1.12 | 0.61 | 0.61 | 0.62 | 0.62 | 0.55 |
| 1ign | 5.19 | **0.51** | **0.51** | **0.51** | **0.51** | 0.68 | 0.68 | 0.68 | **0.51** | 0.51 |
| 1je8 | 8.15 | 0.41 | 0.41 | 0.41 | 0.41 | 0.41 | 0.41 | 0.41 | 0.41 | 0.35 |
| 1jko | 6.55 | 0.66 | 0.66 | 0.66 | 0.66 | 0.76 | 0.62 | **0.38** | **0.38** | 0.38 |
| 1l3l | 4.02 | 0.71 | 0.71 | 0.71 | 0.71 | 0.67 | 0.67 | 0.67 | 0.67 | 0.64 |
| 1lq1 | 5.04 | **0.42** | **0.42** | 0.69 | 0.69 | 0.49 | 0.69 | 0.49 | 0.49 | 0.42 |
| 1mjo | 5.94 | 0.45 | 0.45 | **0.37** | 0.45 | 0.45 | 0.45 | 0.45 | 0.45 | 0.37 |
| 1mnn | 4.46 | 0.96 | **0.56** | **0.56** | **0.56** | 0.92 | 1.00 | **0.56** | **0.56** | 0.56 |
| 1pdn | 6.04 | **0.54** | 1.04 | **0.54** | 0.58 | **0.54** | 0.56 | 0.60 | 0.58 | 0.54 |
| 1per | 4.09 | 0.64 | 0.67 | 0.67 | 0.67 | **0.54** | **0.54** | **0.54** | **0.54** | 0.54 |
| 1qna | 35.70 | 1.05 | 1.05 | 1.05 | 0.54 | 6.66 | 0.54 | 0.54 | 0.54 | 0.49 |
| 1qpi | 5.09 | 0.60 | 0.60 | 0.60 | 0.60 | 0.60 | 0.60 | 0.60 | 0.60 | 0.59 |
| 1qpz | 8.53 | **0.20** | **0.20** | **0.20** | **0.20** | **0.20** | **0.20** | **0.20** | **0.20** | 0.20 |
| 1skn | 5.96 | 0.79 | 0.79 | 0.79 | 0.79 | 0.51 | 0.51 | 0.51 | 0.51 | 0.50 |
| 1tc3 | 7.30 | 1.30 | 1.30 | 0.91 | 0.69 | 0.82 | 0.69 | 0.82 | 0.69 | 0.59 |
| 1tro | 5.02 | **0.55** | **0.55** | **0.55** | **0.55** | **0.55** | **0.55** | **0.55** | **0.55** | 0.55 |
| 1zme | 6.84 | 0.96 | 0.96 | 0.96 | **0.50** | 20.80 | 1.20 | 0.96 | **0.50** | 0.50 |
| 2bop | 6.28 | 0.62 | 0.62 | 0.62 | 0.62 | 0.62 | 0.62 | 0.62 | 0.62 | 0.50 |
| 2cgp | 7.84 | 0.73 | **0.31** | 0.59 | **0.31** | 0.35 | 0.35 | **0.31** | 0.35 | 0.31 |
| 2dgc | 5.75 | **0.06** | **0.06** | **0.06** | **0.06** | **0.06** | **0.06** | **0.06** | **0.06** | 0.06 |
| 2hdd | 5.61 | 0.52 | 0.52 | 0.52 | 0.99 | 0.55 | 0.55 | 0.55 | 0.55 | 0.41 |
| 3bam | 3.77 | 0.44 | **0.42** | 0.44 | 0.44 | **0.42** | **0.42** | **0.42** | 0.44 | 0.42 |
| 3hts | 3.87 | **0.69** | **0.69** | **0.69** | **0.69** | **0.69** | **0.69** | **0.69** | **0.69** | 0.69 |
| 3pvi | 5.71 | 0.70 | 0.78 | 0.87 | 0.64 | 0.63 | **0.58** | 0.64 | 0.64 | 0.58 |
| 6pax | 4.73 | 0.65 | 0.65 | 1.08 | 1.08 | 1.08 | 1.08 | 1.24 | 1.08 | 0.44 |
|  | Median^d^ | 0.54 | 0.53 | 0.56 | 0.54 | 0.55 | 0.54 | 0.54 | 0.53 | 0.44 |
|  | Success^e^ | 15 | 16 | 14 | 14 | 14 | 15 | 16 | 17 | 45 |

This tables using 2000 lowest-RMSD docking decoys as described before, shows the lowest energy structure’s RMSD to the native structures.

^a^Protein data bank identification code.

^b^The degree of overall DNA deformation.

^c^The lowest RMSD decoy.

^d^The median value of the lowest rmsd structure in top five decoys ranked by various energy functions. ^e^How many decoy set successful discriminated the lowest RMSD structures.
