# Supplementary material for: A Structural-Based Strategy for Recognition of Transcription Factor Binding Sites
Source: PLoS One. 2013 Jan 8;8(1):e52460. doi: 10.1371/journal.pone.0052460 (PMC3540023; doi:10.1371/journal.pone.0052460)
Supplement: Table S3 — PDB list for predict TFBS. (DOC) [file pone.0052460.s003.doc]

**Table S3. PDB list for predict TFBS**

| Training Set for predict TFBS  (PDB id) | 3l2c 1a3q 1a73 1am9 1b01 1b3t 1bc8 1bdt 1bf5 1bl0 1cez 1cf7 1ckt 1cl8 1cw0 1d02 1dc1 1dct 1dew 1dfm 1diz 1dsz 1e3o 1efa 1egw 1emh 1ewn 1f4k 1fiu 1flo 1fok 1fyl 1gd2 1gdt 1gu4 1gxp 1h6f 1h9d 1hlv 1i3j 1iaw 1ic8 1j1v 1j3e 1jb7 1je8 1jey 1jj4 1jko 1jnm 1jt0 1jx4 1k3x 1k4t 1ku7 1kx5 1l3l 1lmb 1lq1 1m3q 1mdy 1mjo 1mtl 1mus 1nkp 1odh 1oe4 1omh 1orn 1oup 1owf 1ozj 1p71 1p7h 1p8k 1pp7 1qna 1qpi 1qpz 1qrv 1qzh 1r2z 1r71 1r8d 1r8e 1rep 1rh6 1rrq 1rxw 1rzr 1sa3 1skn 1sx5 1sxq 1t05 1t9i 1tc3 1tez 1tro 1u3e 1u8b 1ubd 1uut 1v15 1w0u 1wb9 1wte 1x9m 1x9n 1xo0 1xpx 1xsd 1xyi 1y8z 1yf3 1z19 1z63 1z9c 1zrf 1zs4 1ztw 1zx4 2a3v 2ac0 2aor 2aq4 2bgw 2bnw 2bsq 2bzf 2c5r 2c7p 2c9l 2dnj 2dp6 2dpi 2drp 2dtu 2e1c 2e52 2er8 2ex5 2ezv 2fcc 2fio 2fkc 2fmp 2fr4 2g1p 2gb7 2gig 2h27 2h7g 2hdd 2heo 2hhv 2i06 2ih2 2ihm 2ihn 2irf 2is6 2isz 2nq9 2ntc 2o4a 2o8b 2oaa 2odi 2ofi 2owo 2p0j 2p5l 2pyj 2qhb 2qnf 2qsh 2r1j 2r9l 2rba 2rbf 2rgr 2vjv 2vla 2yvh 2z3x 2zhg 3bam 3bep 3bkz 3brg 3bs1 3btx 3c0w 3c25 3c2i 3clc 3clz 3cro 3dfx 3dvo 3pvi 6cro 6pax |
| --- | --- |
| All 212 complexes  (PDB id) | 3l2c 1a3q 1a73 1am9 1b01 1b3t 1bc8 1bdt 1bf5 1bl0 1cez 1cf7 1ckt 1cl8 1cw0 1d02 1dc1 1dct 1dew 1dfm 1diz 1dsz 1e3o 1efa 1egw 1emh 1ewn 1f4k 1fiu 1flo 1fok 1fyl 1gd2 1gdt 1gu4 1gxp 1h6f 1h9d 1hlv 1i3j 1iaw 1ic8 1j1v 1j3e 1jb7 1je8 1jey 1jj4 1jko 1jnm 1jt0 1jx4 1k3x 1k4t 1ku7 1kx5 1l3l 1lmb 1lq1 1m3q 1mdy 1mjo 1mtl 1mus 1nkp 1odh 1oe4 1omh 1orn 1oup 1owf 1ozj 1p71 1p7h 1p8k 1pp7 1qna 1qpi 1qpz 1qrv 1qzh 1r2z 1r71 1r8d 1r8e 1rep 1rh6 1rrq 1rxw 1rzr 1sa3 1skn 1sx5 1sxq 1t05 1t9i 1tc3 1tez 1tro 1u3e 1u8b 1ubd 1uut 1v15 1w0u 1wb9 1wte 1x9m 1x9n 1xo0 1xpx 1xsd 1xyi 1y8z 1yf3 1z19 1z63 1z9c 1zrf 1zs4 1ztw 1zx4 2a3v 2ac0 2aor 2aq4 2bgw 2bnw 2bsq 2bzf 2c5r 2c7p 2c9l 2dnj 2dp6 2dpi 2drp 2dtu 2e1c 2e52 2er8 2ex5 2ezv 2fcc 2fio 2fkc 2fmp 2fr4 2g1p 2gb7 2gig 2h27 2h7g 2hdd 2heo 2hhv 2i06 2ih2 2ihm 2ihn 2irf 2is6 2isz 2nq9 2ntc 2o4a 2o8b 2oaa 2odi 2ofi 2owo 2p0j 2p5l 2pyj 2qhb 2qnf 2qsh 2r1j 2r9l 2rba 2rbf 2rgr 2vjv 2vla 2yvh 2z3x 2zhg 3bam 3bep 3bkz 3brg 3bs1 3btx 3c0w 3c25 3c2i 3clc 3clz 3cro 3dfx 3dvo 3pvi 6cro 6pax |
