# Supplementary material for: A Structural-Based Strategy for Recognition of Transcription Factor Binding Sites
Source: PLoS One. 2013 Jan 8;8(1):e52460. doi: 10.1371/journal.pone.0052460 (PMC3540023; doi:10.1371/journal.pone.0052460)
Supplement: Table S4 — RMSD list for Different Structure Templates. (DOC) [file pone.0052460.s004.doc]

**Table S4. RMSD list for Different Structure Templates**

| TF Name | RMSD Group | | | |
| --- | --- | --- | --- | --- |
|  | 1 | 2 | 3 | 4 |
| GAL4 | 0.955 | 1.905 | 2.858 | 3.935 |
| GCN4 | 0.828 | 1.991 | 2.98 | 3.918 |
| HAP1 | 0.915 | 1.831 | 2.941 | 3.966 |
| LEU3 | 0.929 | 1.976 | 2.892 | 3.639 |
| MATA1 | 0.872 | 1.968 | 2.855 | 3.965 |
| MATALPHA2 | 0.973 | 1.99 | 2.994 | 3.263 |
| MCM1 | 0.958 | 1.888 | 2.987 | 3.85 |
| MCM1_MATALPHA2 | 0.958 | 1.888 | 2.987 | 3.85 |
| NDT80 | 0.949 | 1.939 | 2.996 | 3.933 |
| PHO4 | 0.936 | 1.99 | 3 | 3.959 |
| PPR1 | 0.882 | 1.96 | 2.887 | 3.938 |
| PUT3 | 0.953 | 1.998 | 2.976 | 3.9 |
| RAP1 | 0.943 | 1.93 | 2.832 | 3.885 |
| TBP | 0.945 | 1.971 | 2.875 | 3.418 |
| TFIIA | 0.993 | 1.999 | 2.912 | 3.012 |
| TFIIA_TBP | 0.993 | 1.999 | 2.912 | 3.012 |
