# Supplementary material for: A Structural-Based Strategy for Recognition of Transcription Factor Binding Sites
Source: PLoS One. 2013 Jan 8;8(1):e52460. doi: 10.1371/journal.pone.0052460 (PMC3540023; doi:10.1371/journal.pone.0052460)
Supplement: Table S5 — Training set for Yeast_Self_Mutant. Nmutated bases have been mutated from Nsequence in Yeast_Self_Mutant set. (DOC) [file pone.0052460.s005.doc]

**Table S5. Training set for Yeast_Self_Mutant**

N_mutated_ bases have been mutated from N_sequence_ in Yeast_Self_Mutant set.

| TF Name | N Mutated | N Sequence | Mutated | | | | Sequence Mutated to | Original Sequence |
| --- | --- | --- | --- | --- | --- | --- | --- | --- |
|  |  |  | A | C | G | T |  |  |
| GAL4 | 14 | 19 | 3 | 5 | 6 | 5 | TGCGTTCCAAAGGCTGTCG | CCGGAGGACAGTCCTCCGG |
| GCN4 | 14 | 18 | 4 | 5 | 3 | 6 | TGTGCAACACCTACTGTT | TCCTATGACTCATCCAGT |
| HAP1 | 15 | 20 | 5 | 4 | 9 | 2 | AAGACCGTGAGGGTAGCGCG | GCGCTATTATCGCTATTAGC |
| LEU3 | 11 | 14 | 3 | 2 | 3 | 6 | GTACGATTTTACGT | TGCCGGTACCGGCA |
| MATA1 | 12 | 19 | 7 | 4 | 4 | 4 | TAGATCATGCGTAGAAACC | CATGTAATTTATTACATCA |
| MATALPHA2 | 18 | 20 | 3 | 8 | 2 | 7 | CCATCTGACTCCCGTTTTAC | CATGTAATTCATTTACACGC |
| MCM1_MATALPHA2 | 17 | 25 | 6 | 6 | 7 | 6 | GCAGCGTCTTCGAGAACGCGATTAT | ATTACCTAATAGGGAAATTTACACG |
| MCM10 | 17 | 25 | 7 | 7 | 7 | 4 | AGCAAGCCGCTTAGGACATTAGGCC | ATTACCTAATAGGGAAATTTACACG |
| NDT80 | 12 | 13 | 2 | 3 | 3 | 5 | CAGTATGCTTGCT | GCGACACAAAAAC |
| PHO4 | 16 | 17 | 2 | 4 | 5 | 6 | TCTGATTGGTATCGGCC | CTCACACGTGGGACTAG |
| PPR1 | 12 | 14 | 4 | 3 | 4 | 3 | AAAGGTGGCTCTAC | TCGGCAATTGCCGA |
| PUT3 | 10 | 14 | 7 | 3 | 4 | 0 | AGGCGAACCAAAAG | CGGGAGCCACTCCG |
| RAP1 | 11 | 18 | 1 | 4 | 10 | 3 | CGCAGGTTCGGTCGGGGG | CGCACACCCACACACCAG |
| TBP | 9 | 13 | 2 | 3 | 4 | 4 | GTTGAACTGGCCT | GTATATAAAACGG |
| TFIIA_TBP | 11 | 16 | 2 | 3 | 4 | 7 | TGAGCATTGTTGCTTC | TGTATGTATATAAAAC |
| TFIIA | 14 | 16 | 2 | 6 | 3 | 5 | CATCACCTTTGGGCCT | TGTATGTATATAAAAC |
| Sum Total | 213 | 281 | 60 | 70 | 78 | 73 |  |  |
