# Supplementary material for: A Structural-Based Strategy for Recognition of Transcription Factor Binding Sites
Source: PLoS One. 2013 Jan 8;8(1):e52460. doi: 10.1371/journal.pone.0052460 (PMC3540023; doi:10.1371/journal.pone.0052460)
Supplement: Table S6 — Prediction Results. (DOC) [file pone.0052460.s006.doc]

**Table S6. Prediction Results**

Yeast_Self

| RMSD Group | Transcription Factor | TP | FN | FP | Sens | Spec | Cutoff |
| --- | --- | --- | --- | --- | --- | --- | --- |
| 0 | GAL4 | 12 | 3 | 0 | 0.80 | 1.00 | -232.39 |
| 0 | GCN4 | 7 | 11 | 2 | 0.39 | 0.78 | -251.82 |
| 0 | HAP1 | 8 | 1 | 56 | 0.89 | 0.12 | -198.32 |
| 0 | LEU3 | 5 | 0 | 0 | 1.00 | 1.00 | -411.40 |
| 0 | MATA1 | 1 | 0 | 0 | 1.00 | 1.00 | -142.58 |
| 0 | MATALPHA2 | 5 | 5 | 1 | 0.50 | 0.83 | -182.38 |
| 0 | MCM1_MATALPHA2 | 1 | 0 | 0 | 1.00 | 1.00 | -462.55 |
| 0 | MCM1 | 25 | 1 | 26 | 0.96 | 0.49 | -220.85 |
| 0 | NDT80 | 1 | 0 | 0 | 1.00 | 1.00 | -353.70 |
| 0 | PHO4 | 4 | 0 | 10 | 1.00 | 0.29 | -224.57 |
| 0 | PPR1 | 1 | 0 | 0 | 1.00 | 1.00 | -203.10 |
| 0 | PUT3 | 2 | 1 | 0 | 0.67 | 1.00 | -236.63 |
| 0 | RAP1 | 3 | 21 | 0 | 0.12 | 1.00 | -498.91 |
| 0 | TBP | 5 | 2 | 1 | 0.71 | 0.83 | -388.57 |
| 0 | TFIIA_TBP | 1 | 0 | 1 | 1.00 | 0.50 | -330.22 |
| 0 | TFIIA | 1 | 0 | 6 | 1.00 | 0.14 | -30.43 |
| 1 | GAL4 | 11 | 4 | 0 | 0.73 | 1.00 | -210.94 |
| 1 | GCN4 | 7 | 11 | 2 | 0.39 | 0.78 | -236.81 |
| 1 | HAP1 | 8 | 1 | 50 | 0.89 | 0.14 | -185.78 |
| 1 | LEU3 | 5 | 0 | 0 | 1.00 | 1.00 | -358.33 |
| 1 | MATA1 | 1 | 0 | 0 | 1.00 | 1.00 | -158.29 |
| 1 | MATALPHA2 | 5 | 5 | 1 | 0.50 | 0.83 | -169.06 |
| 1 | MCM1_MATALPHA2 | 1 | 0 | 0 | 1.00 | 1.00 | -435.42 |
| 1 | MCM1 | 26 | 0 | 50 | 1.00 | 0.34 | -182.15 |
| 1 | NDT80 | 1 | 0 | 0 | 1.00 | 1.00 | -404.66 |
| 1 | PHO4 | 1 | 3 | 0 | 0.25 | 1.00 | -281.37 |
| 1 | PPR1 | 1 | 0 | 0 | 1.00 | 1.00 | -181.87 |
| 1 | PUT3 | 1 | 2 | 0 | 0.33 | 1.00 | -209.71 |
| 1 | RAP1 | 19 | 5 | 70 | 0.79 | 0.21 | -359.93 |
| 1 | TBP | 5 | 2 | 3 | 0.71 | 0.62 | -332.20 |
| 1 | TFIIA_TBP | 1 | 0 | 1 | 1.00 | 0.50 | -340.82 |
| 1 | TFIIA | 1 | 0 | 6 | 1.00 | 0.14 | -32.83 |
| 2 | GAL4 | 11 | 4 | 0 | 0.73 | 1.00 | -248.73 |
| 2 | GCN4 | 7 | 11 | 2 | 0.39 | 0.78 | -247.00 |
| 2 | HAP1 | 8 | 1 | 55 | 0.89 | 0.13 | -199.36 |
| 2 | LEU3 | 5 | 0 | 0 | 1.00 | 1.00 | -395.40 |
| 2 | MATA1 | 1 | 0 | 0 | 1.00 | 1.00 | -172.44 |
| 2 | MATALPHA2 | 3 | 7 | 0 | 0.30 | 1.00 | -173.72 |
| 2 | MCM1_MATALPHA2 | 1 | 0 | 0 | 1.00 | 1.00 | -428.60 |
| 2 | MCM1 | 26 | 0 | 49 | 1.00 | 0.35 | -179.78 |
| 2 | NDT80 | 1 | 0 | 0 | 1.00 | 1.00 | -456.66 |
| 2 | PHO4 | 1 | 3 | 0 | 0.25 | 1.00 | -291.87 |
| 2 | PPR1 | 1 | 0 | 0 | 1.00 | 1.00 | -152.63 |
| 2 | PUT3 | 3 | 0 | 28 | 1.00 | 0.10 | -160.36 |
| 2 | RAP1 | 14 | 10 | 20 | 0.58 | 0.41 | -421.11 |
| 2 | TBP | 3 | 4 | 0 | 0.43 | 1.00 | -353.05 |
| 2 | TFIIA_TBP | 1 | 0 | 1 | 1.00 | 0.50 | -387.44 |
| 2 | TFIIA | 1 | 0 | 4 | 1.00 | 0.20 | -34.59 |
| 3 | GAL4 | 14 | 1 | 4 | 0.93 | 0.78 | -276.07 |
| 3 | GCN4 | 7 | 11 | 2 | 0.39 | 0.78 | -264.46 |
| 3 | HAP1 | 8 | 1 | 57 | 0.89 | 0.12 | -199.20 |
| 3 | LEU3 | 5 | 0 | 0 | 1.00 | 1.00 | -456.76 |
| 3 | MATA1 | 1 | 0 | 0 | 1.00 | 1.00 | -185.99 |
| 3 | MATALPHA2 | 3 | 7 | 0 | 0.30 | 1.00 | -175.04 |
| 3 | MCM1_MATALPHA2 | 1 | 0 | 0 | 1.00 | 1.00 | -427.85 |
| 3 | MCM1 | 25 | 1 | 33 | 0.96 | 0.43 | -180.97 |
| 3 | NDT80 | 1 | 0 | 0 | 1.00 | 1.00 | -557.58 |
| 3 | PHO4 | 1 | 3 | 0 | 0.25 | 1.00 | -294.05 |
| 3 | PPR1 | 1 | 0 | 1 | 1.00 | 0.50 | -134.92 |
| 3 | PUT3 | 2 | 1 | 1 | 0.67 | 0.67 | -218.61 |
| 3 | RAP1 | 14 | 10 | 14 | 0.58 | 0.50 | -435.78 |
| 3 | TBP | 3 | 4 | 0 | 0.43 | 1.00 | -388.60 |
| 3 | TFIIA_TBP | 1 | 0 | 2 | 1.00 | 0.33 | -472.21 |
| 3 | TFIIA | 1 | 0 | 4 | 1.00 | 0.20 | -40.96 |
| 4 | GAL4 | 14 | 1 | 6 | 0.93 | 0.70 | -299.38 |
| 4 | GCN4 | 7 | 11 | 1 | 0.39 | 0.88 | -304.58 |
| 4 | HAP1 | 8 | 1 | 64 | 0.89 | 0.11 | -178.44 |
| 4 | LEU3 | 5 | 0 | 0 | 1.00 | 1.00 | -502.50 |
| 4 | MATA1 | 1 | 0 | 0 | 1.00 | 1.00 | -195.17 |
| 4 | MATALPHA2 | 3 | 7 | 0 | 0.30 | 1.00 | -175.80 |
| 4 | MCM1_MATALPHA2 | 1 | 0 | 0 | 1.00 | 1.00 | -424.83 |
| 4 | MCM1 | 25 | 1 | 32 | 0.96 | 0.44 | -172.92 |
| 4 | NDT80 | 1 | 0 | 0 | 1.00 | 1.00 | -617.94 |
| 4 | PHO4 | 1 | 3 | 0 | 0.25 | 1.00 | -311.22 |
| 4 | PPR1 | 1 | 0 | 7 | 1.00 | 0.12 | -105.71 |
| 4 | PUT3 | 1 | 2 | 0 | 0.33 | 1.00 | -232.34 |
| 4 | RAP1 | 1 | 23 | 0 | 0.04 | 1.00 | -568.51 |
| 4 | TBP | 3 | 4 | 0 | 0.43 | 1.00 | -445.50 |
| 4 | TFIIA_TBP | 1 | 0 | 1 | 1.00 | 0.50 | -515.76 |
| 4 | TFIIA | 1 | 0 | 4 | 1.00 | 0.20 | -49.18 |

Yeast_Native

| RMSD  Group | Transcription Factor | TP | FN | FP | Sens | Spec | Cutoff |
| --- | --- | --- | --- | --- | --- | --- | --- |
| 0 | GAL4 | 13 | 2 | 0 | 0.87 | 1.00 | -170.66 |
| 0 | GCN4 | 7 | 11 | 2 | 0.39 | 0.78 | -131.58 |
| 0 | HAP1 | 9 | 0 | 84 | 1.00 | 0.10 | -85.36 |
| 0 | LEU3 | 5 | 0 | 0 | 1.00 | 1.00 | -320.33 |
| 0 | MATA1 | 1 | 0 | 0 | 1.00 | 1.00 | -88.39 |
| 0 | MATALPHA2 | 5 | 5 | 0 | 0.50 | 1.00 | -130.78 |
| 0 | MCM1_MATALPHA2 | 1 | 0 | 0 | 1.00 | 1.00 | -263.25 |
| 0 | MCM1 | 25 | 1 | 26 | 0.96 | 0.49 | -103.67 |
| 0 | NDT80 | 1 | 0 | 0 | 1.00 | 1.00 | -223.73 |
| 0 | PHO4 | 4 | 0 | 10 | 1.00 | 0.29 | -115.40 |
| 0 | PPR1 | 1 | 0 | 0 | 1.00 | 1.00 | -133.48 |
| 0 | PUT3 | 1 | 2 | 0 | 0.33 | 1.00 | -172.13 |
| 0 | RAP1 | 4 | 20 | 0 | 0.17 | 1.00 | -327.83 |
| 0 | TBP | 5 | 2 | 1 | 0.71 | 0.83 | -334.20 |
| 0 | TFIIA_TBP | 1 | 0 | 1 | 1.00 | 0.50 | -287.44 |
| 0 | TFIIA | 1 | 0 | 7 | 1.00 | 0.12 | -11.62 |
| 1 | GAL4 | 8 | 7 | 0 | 0.53 | 1.00 | -51.78 |
| 1 | GCN4 | 17 | 1 | 114 | 0.94 | 0.13 | -20.97 |
| 1 | HAP1 | 9 | 0 | 20 | 1.00 | 0.31 | -14.14 |
| 1 | LEU3 | 5 | 0 | 0 | 1.00 | 1.00 | -119.92 |
| 1 | MATA1 | 1 | 0 | 0 | 1.00 | 1.00 | -61.65 |
| 1 | MATALPHA2 | 8 | 2 | 7 | 0.80 | 0.53 | -55.82 |
| 1 | MCM1_MATALPHA2 | 1 | 0 | 0 | 1.00 | 1.00 | -40.10 |
| 1 | MCM1 | 12 | 14 | 3 | 0.46 | 0.80 | -17.30 |
| 1 | NDT80 | 1 | 0 | 0 | 1.00 | 1.00 | -58.90 |
| 1 | PHO4 | 4 | 0 | 8 | 1.00 | 0.33 | 6.43 |
| 1 | PPR1 | 1 | 0 | 0 | 1.00 | 1.00 | -44.03 |
| 1 | PUT3 | 1 | 2 | 0 | 0.33 | 1.00 | -21.28 |
| 1 | RAP1 | 20 | 4 | 80 | 0.83 | 0.20 | -27.11 |
| 1 | TBP | 5 | 2 | 3 | 0.71 | 0.62 | -38.32 |
| 1 | TFIIA_TBP | 1 | 0 | 0 | 1.00 | 1.00 | -140.91 |
| 1 | TFIIA | 1 | 0 | 6 | 1.00 | 0.14 | -4.64 |
| 2 | GAL4 | 15 | 0 | 55 | 1.00 | 0.21 | 25.48 |
| 2 | GCN4 | 17 | 1 | 121 | 0.94 | 0.12 | 39.26 |
| 2 | HAP1 | 9 | 0 | 36 | 1.00 | 0.20 | 60.78 |
| 2 | LEU3 | 5 | 0 | 16 | 1.00 | 0.24 | 33.98 |
| 2 | MATA1 | 1 | 0 | 0 | 1.00 | 1.00 | -3.40 |
| 2 | MATALPHA2 | 2 | 8 | 0 | 0.20 | 1.00 | 15.62 |
| 2 | MCM1_MATALPHA2 | 1 | 0 | 0 | 1.00 | 1.00 | 80.72 |
| 2 | MCM1 | 20 | 6 | 130 | 0.77 | 0.13 | 43.35 |
| 2 | NDT80 | 1 | 0 | 0 | 1.00 | 1.00 | 33.47 |
| 2 | PHO4 | 3 | 1 | 2 | 0.75 | 0.60 | 63.80 |
| 2 | PPR1 | 1 | 0 | 8 | 1.00 | 0.11 | 19.85 |
| 2 | PUT3 | 3 | 0 | 32 | 1.00 | 0.09 | 44.21 |
| 2 | RAP1 | 13 | 11 | 30 | 0.54 | 0.30 | 20.75 |
| 2 | TBP | 7 | 0 | 114 | 1.00 | 0.06 | 63.22 |
| 2 | TFIIA_TBP | 1 | 0 | 0 | 1.00 | 1.00 | 13.55 |
| 2 | TFIIA | 1 | 0 | 15 | 1.00 | 0.06 | 5.41 |
| 3 | GAL4 | 15 | 0 | 88 | 1.00 | 0.15 | 75.73 |
| 3 | GCN4 | 10 | 8 | 78 | 0.56 | 0.11 | 61.00 |
| 3 | HAP1 | 1 | 8 | 0 | 0.11 | 1.00 | 90.26 |
| 3 | LEU3 | 4 | 1 | 109 | 0.80 | 0.04 | 115.86 |
| 3 | MATA1 | 1 | 0 | 0 | 1.00 | 1.00 | 18.96 |
| 3 | MATALPHA2 | 1 | 9 | 0 | 0.10 | 1.00 | 23.70 |
| 3 | MCM1_MATALPHA2 | 1 | 0 | 6 | 1.00 | 0.14 | 137.07 |
| 3 | MCM1 | 13 | 12 | 139 | 0.52 | 0.09 | 68.43 |
| 3 | NDT80 | 1 | 0 | 17 | 1.00 | 0.06 | 137.19 |
| 3 | PHO4 | 4 | 0 | 30 | 1.00 | 0.12 | 80.00 |
| 3 | PPR1 | 1 | 0 | 31 | 1.00 | 0.03 | 31.88 |
| 3 | PUT3 | 3 | 0 | 33 | 1.00 | 0.08 | 71.48 |
| 3 | RAP1 | 15 | 8 | 22 | 0.65 | 0.41 | 81.86 |
| 3 | TBP | 6 | 1 | 115 | 0.86 | 0.05 | 79.85 |
| 3 | TFIIA_TBP | 1 | 0 | 19 | 1.00 | 0.05 | 130.56 |
| 3 | TFIIA | 1 | 0 | 32 | 1.00 | 0.03 | 7.30 |
| 4 | GAL4 | 15 | 0 | 126 | 1.00 | 0.11 | 94.92 |
| 4 | GCN4 | 12 | 6 | 117 | 0.67 | 0.09 | 89.30 |
| 4 | HAP1 | 9 | 0 | 113 | 1.00 | 0.07 | 98.72 |
| 4 | LEU3 | 0 | 5 | 160 | 0.00 | 0.00 | 9999.99 |
| 4 | MATA1 | 1 | 0 | 11 | 1.00 | 0.08 | 37.22 |
| 4 | MATALPHA2 | 1 | 7 | 0 | 0.12 | 1.00 | 31.20 |
| 4 | MCM1_MATALPHA2 | 1 | 0 | 6 | 1.00 | 0.14 | 157.95 |
| 4 | MCM1 | 15 | 10 | 124 | 0.60 | 0.11 | 76.95 |
| 4 | NDT80 | 1 | 0 | 20 | 1.00 | 0.05 | 163.36 |
| 4 | PHO4 | 4 | 0 | 35 | 1.00 | 0.10 | 99.65 |
| 4 | PPR1 | 1 | 0 | 13 | 1.00 | 0.07 | 34.80 |
| 4 | PUT3 | 3 | 0 | 21 | 1.00 | 0.12 | 75.35 |
| 4 | RAP1 | 20 | 4 | 103 | 0.83 | 0.16 | 128.05 |
| 4 | TBP | 4 | 3 | 44 | 0.57 | 0.08 | 104.43 |
| 4 | TFIIA_TBP | 1 | 0 | 22 | 1.00 | 0.04 | 152.34 |
| 4 | TFIIA | 1 | 0 | 5 | 1.00 | 0.17 | 6.14 |

PDB_Native

| RMSD  Group | Transcription Factor | TP | FN | FP | Sens | Spec | Cutoff |
| --- | --- | --- | --- | --- | --- | --- | --- |
| 0 | GAL4 | 13 | 2 | 0 | 0.87 | 1.00 | -52.72 |
| 0 | GCN4 | 12 | 6 | 12 | 0.67 | 0.50 | -49.09 |
| 0 | HAP1 | 9 | 0 | 38 | 1.00 | 0.19 | -26.66 |
| 0 | LEU3 | 5 | 0 | 0 | 1.00 | 1.00 | -65.62 |
| 0 | MATA1 | 1 | 0 | 1 | 1.00 | 0.50 | -21.03 |
| 0 | MATALPHA2 | 10 | 0 | 135 | 1.00 | 0.07 | -14.30 |
| 0 | MCM1_MATALPHA2 | 1 | 0 | 0 | 1.00 | 1.00 | -62.75 |
| 0 | MCM1 | 24 | 2 | 116 | 0.92 | 0.17 | -26.97 |
| 0 | NDT80 | 1 | 0 | 0 | 1.00 | 1.00 | -79.81 |
| 0 | PHO4 | 1 | 3 | 0 | 0.25 | 1.00 | -49.30 |
| 0 | PPR1 | 1 | 0 | 0 | 1.00 | 1.00 | -6.89 |
| 0 | PUT3 | 1 | 2 | 0 | 0.33 | 1.00 | -56.88 |
| 0 | RAP1 | 15 | 9 | 8 | 0.62 | 0.65 | -78.05 |
| 0 | TBP | 6 | 1 | 41 | 0.86 | 0.13 | -40.42 |
| 0 | TFIIA_TBP | 1 | 0 | 3 | 1.00 | 0.25 | -45.92 |
| 0 | TFIIA | 1 | 0 | 20 | 1.00 | 0.05 | -2.86 |
| 1 | GAL4 | 10 | 5 | 3 | 0.67 | 0.77 | 9.42 |
| 1 | GCN4 | 1 | 17 | 0 | 0.06 | 1.00 | -41.22 |
| 1 | HAP1 | 8 | 1 | 14 | 0.89 | 0.36 | -11.32 |
| 1 | LEU3 | 5 | 0 | 0 | 1.00 | 1.00 | -7.55 |
| 1 | MATA1 | 1 | 0 | 1 | 1.00 | 0.50 | -13.36 |
| 1 | MATALPHA2 | 9 | 1 | 116 | 0.90 | 0.07 | -1.34 |
| 1 | MCM1_MATALPHA2 | 1 | 0 | 0 | 1.00 | 1.00 | -26.30 |
| 1 | MCM1 | 19 | 7 | 127 | 0.73 | 0.13 | -14.75 |
| 1 | NDT80 | 1 | 0 | 0 | 1.00 | 1.00 | -24.04 |
| 1 | PHO4 | 1 | 3 | 0 | 0.25 | 1.00 | -7.92 |
| 1 | PPR1 | 1 | 0 | 0 | 1.00 | 1.00 | 7.53 |
| 1 | PUT3 | 1 | 2 | 0 | 0.33 | 1.00 | 1.15 |
| 1 | RAP1 | 15 | 9 | 13 | 0.62 | 0.54 | -14.02 |
| 1 | TBP | 7 | 0 | 67 | 1.00 | 0.09 | 3.07 |
| 1 | TFIIA_TBP | 1 | 0 | 2 | 1.00 | 0.33 | -15.47 |
| 1 | TFIIA | 1 | 0 | 2 | 1.00 | 0.33 | -1.17 |
| 2 | GAL4 | 14 | 1 | 35 | 0.93 | 0.29 | 77.99 |
| 2 | GCN4 | 1 | 17 | 0 | 0.06 | 1.00 | -2.52 |
| 2 | HAP1 | 9 | 0 | 100 | 1.00 | 0.08 | 38.22 |
| 2 | LEU3 | 5 | 0 | 3 | 1.00 | 0.62 | 70.00 |
| 2 | MATA1 | 1 | 0 | 2 | 1.00 | 0.33 | 1.56 |
| 2 | MATALPHA2 | 8 | 1 | 85 | 0.89 | 0.09 | 20.80 |
| 2 | MCM1_MATALPHA2 | 1 | 0 | 9 | 1.00 | 0.10 | 42.88 |
| 2 | MCM1 | 19 | 7 | 114 | 0.73 | 0.14 | 8.72 |
| 2 | NDT80 | 1 | 0 | 0 | 1.00 | 1.00 | 20.88 |
| 2 | PHO4 | 4 | 0 | 19 | 1.00 | 0.17 | 46.07 |
| 2 | PPR1 | 1 | 0 | 1 | 1.00 | 0.50 | 15.30 |
| 2 | PUT3 | 3 | 0 | 37 | 1.00 | 0.07 | 59.75 |
| 2 | RAP1 | 1 | 23 | 0 | 0.04 | 1.00 | 19.67 |
| 2 | TBP | 6 | 1 | 129 | 0.86 | 0.04 | 38.48 |
| 2 | TFIIA_TBP | 1 | 0 | 10 | 1.00 | 0.09 | 60.05 |
| 2 | TFIIA | 1 | 0 | 12 | 1.00 | 0.08 | 2.16 |
| 3 | GAL4 | 14 | 1 | 56 | 0.93 | 0.20 | 140.45 |
| 3 | GCN4 | 12 | 6 | 127 | 0.67 | 0.09 | 30.83 |
| 3 | HAP1 | 8 | 1 | 29 | 0.89 | 0.22 | 72.10 |
| 3 | LEU3 | 4 | 1 | 144 | 0.80 | 0.03 | 136.67 |
| 3 | MATA1 | 1 | 0 | 5 | 1.00 | 0.17 | 15.30 |
| 3 | MATALPHA2 | 8 | 1 | 117 | 0.89 | 0.06 | 28.54 |
| 3 | MCM1_MATALPHA2 | 1 | 0 | 22 | 1.00 | 0.04 | 94.32 |
| 3 | MCM1 | 1 | 25 | 0 | 0.04 | 1.00 | 30.72 |
| 3 | NDT80 | 1 | 0 | 1 | 1.00 | 0.50 | 93.11 |
| 3 | PHO4 | 4 | 0 | 34 | 1.00 | 0.11 | 73.49 |
| 3 | PPR1 | 1 | 0 | 22 | 1.00 | 0.04 | 21.38 |
| 3 | PUT3 | 3 | 0 | 11 | 1.00 | 0.21 | 85.76 |
| 3 | RAP1 | 17 | 7 | 89 | 0.71 | 0.16 | 62.84 |
| 3 | TBP | 5 | 2 | 84 | 0.71 | 0.06 | 72.86 |
| 3 | TFIIA_TBP | 1 | 0 | 2 | 1.00 | 0.33 | 170.35 |
| 3 | TFIIA | 1 | 0 | 8 | 1.00 | 0.11 | 3.55 |
| 4 | GAL4 | 11 | 3 | 110 | 0.79 | 0.09 | 173.79 |
| 4 | GCN4 | 6 | 12 | 152 | 0.33 | 0.04 | 75.47 |
| 4 | HAP1 | 9 | 0 | 117 | 1.00 | 0.07 | 79.30 |
| 4 | LEU3 | 4 | 1 | 125 | 0.80 | 0.03 | 179.42 |
| 4 | MATA1 | 1 | 0 | 3 | 1.00 | 0.25 | 25.61 |
| 4 | MATALPHA2 | 1 | 7 | 0 | 0.12 | 1.00 | 32.70 |
| 4 | MCM1_MATALPHA2 | 1 | 0 | 15 | 1.00 | 0.06 | 125.47 |
| 4 | MCM1 | 13 | 13 | 146 | 0.50 | 0.08 | 52.82 |
| 4 | NDT80 | 1 | 0 | 2 | 1.00 | 0.33 | 143.18 |
| 4 | PHO4 | 4 | 0 | 42 | 1.00 | 0.09 | 118.78 |
| 4 | PPR1 | 1 | 0 | 13 | 1.00 | 0.07 | 23.86 |
| 4 | PUT3 | 3 | 0 | 31 | 1.00 | 0.09 | 101.36 |
| 4 | RAP1 | 11 | 12 | 119 | 0.48 | 0.08 | 105.88 |
| 4 | TBP | 7 | 0 | 35 | 1.00 | 0.17 | 113.87 |
| 4 | TFIIA_TBP | 1 | 0 | 23 | 1.00 | 0.04 | 203.57 |
| 4 | TFIIA | 1 | 0 | 2 | 1.00 | 0.33 | -0.61 |

Yeast_Self_Mutant

| RMSD  Group | Transcription Factor | TP | FN | FP | Sens | Spec | Cutoff |
| --- | --- | --- | --- | --- | --- | --- | --- |
| 0 | GAL4 | 2 | 13 | 0 | 0.13 | 1.00 | -229.16 |
| 0 | GCN4 | 9 | 9 | 126 | 0.50 | 0.07 | -175.91 |
| 0 | HAP1 | 2 | 7 | 0 | 0.22 | 1.00 | -274.18 |
| 0 | LEU3 | 3 | 2 | 130 | 0.60 | 0.02 | -299.40 |
| 0 | MATA1 | 1 | 0 | 12 | 1.00 | 0.08 | -101.97 |
| 0 | MATALPHA2 | 7 | 2 | 148 | 0.78 | 0.05 | 9999.00 |
| 0 | MCM1_MATALPHA2 | 1 | 0 | 20 | 1.00 | 0.05 | -288.62 |
| 0 | MCM1 | 1 | 24 | 1 | 0.04 | 0.50 | -236.47 |
| 0 | NDT80 | 1 | 0 | 35 | 1.00 | 0.03 | -217.11 |
| 0 | PHO4 | 4 | 0 | 29 | 1.00 | 0.12 | -200.06 |
| 0 | PPR1 | 1 | 0 | 16 | 1.00 | 0.06 | -154.92 |
| 0 | PUT3 | 3 | 0 | 139 | 1.00 | 0.02 | -160.76 |
| 0 | RAP1 | 8 | 15 | 126 | 0.35 | 0.06 | -299.36 |
| 0 | TBP | 5 | 2 | 94 | 0.71 | 0.05 | -280.32 |
| 0 | TFIIA_TBP | 1 | 0 | 6 | 1.00 | 0.14 | -317.30 |
| 0 | TFIIA | 1 | 0 | 6 | 1.00 | 0.14 | -28.96 |
| 1 | GAL4 | 13 | 2 | 117 | 0.87 | 0.10 | -78.87 |
| 1 | GCN4 | 10 | 8 | 142 | 0.56 | 0.07 | 9999.99 |
| 1 | HAP1 | 2 | 7 | 0 | 0.22 | 1.00 | -104.28 |
| 1 | LEU3 | 3 | 2 | 74 | 0.60 | 0.04 | -145.96 |
| 1 | MATA1 | 1 | 0 | 13 | 1.00 | 0.07 | -74.75 |
| 1 | MATALPHA2 | 8 | 1 | 142 | 0.89 | 0.05 | -68.88 |
| 1 | MCM1_MATALPHA2 | 1 | 0 | 28 | 1.00 | 0.03 | -86.65 |
| 1 | MCM1 | 1 | 24 | 0 | 0.04 | 1.00 | -88.60 |
| 1 | NDT80 | 1 | 0 | 14 | 1.00 | 0.07 | -94.32 |
| 1 | PHO4 | 4 | 0 | 18 | 1.00 | 0.18 | -50.73 |
| 1 | PPR1 | 1 | 0 | 37 | 1.00 | 0.03 | -46.73 |
| 1 | PUT3 | 3 | 0 | 99 | 1.00 | 0.03 | -39.40 |
| 1 | RAP1 | 8 | 15 | 143 | 0.35 | 0.05 | -104.44 |
| 1 | TBP | 7 | 0 | 124 | 1.00 | 0.05 | -36.41 |
| 1 | TFIIA_TBP | 1 | 0 | 6 | 1.00 | 0.14 | -132.98 |
| 1 | TFIIA | 1 | 0 | 10 | 1.00 | 0.09 | -14.59 |
| 2 | GAL4 | 1 | 13 | 0 | 0.07 | 1.00 | -58.75 |
| 2 | GCN4 | 11 | 7 | 144 | 0.61 | 0.07 | -6.03 |
| 2 | HAP1 | 1 | 8 | 0 | 0.11 | 1.00 | -8.58 |
| 2 | LEU3 | 5 | 0 | 72 | 1.00 | 0.06 | -28.20 |
| 2 | MATA1 | 1 | 0 | 30 | 1.00 | 0.03 | -16.78 |
| 2 | MATALPHA2 | 8 | 1 | 135 | 0.89 | 0.06 | -6.38 |
| 2 | MCM1_MATALPHA2 | 1 | 0 | 9 | 1.00 | 0.10 | -1.05 |
| 2 | MCM1 | 1 | 25 | 0 | 0.04 | 1.00 | -18.97 |
| 2 | NDT80 | 1 | 0 | 7 | 1.00 | 0.12 | -13.78 |
| 2 | PHO4 | 4 | 0 | 23 | 1.00 | 0.15 | 11.70 |
| 2 | PPR1 | 1 | 0 | 5 | 1.00 | 0.17 | -5.59 |
| 2 | PUT3 | 3 | 0 | 63 | 1.00 | 0.05 | 6.12 |
| 2 | RAP1 | 4 | 18 | 145 | 0.18 | 0.03 | -50.02 |
| 2 | TBP | 7 | 0 | 110 | 1.00 | 0.06 | 20.44 |
| 2 | TFIIA_TBP | 1 | 0 | 22 | 1.00 | 0.04 | -15.50 |
| 2 | TFIIA | 1 | 0 | 7 | 1.00 | 0.12 | -1.90 |
| 3 | GAL4 | 12 | 3 | 124 | 0.80 | 0.09 | -6.33 |
| 3 | GCN4 | 8 | 10 | 131 | 0.44 | 0.06 | 8.12 |
| 3 | HAP1 | 3 | 6 | 0 | 0.33 | 1.00 | 27.22 |
| 3 | LEU3 | 5 | 0 | 57 | 1.00 | 0.08 | 35.83 |
| 3 | MATA1 | 1 | 0 | 43 | 1.00 | 0.02 | -3.00 |
| 3 | MATALPHA2 | 10 | 0 | 135 | 1.00 | 0.07 | -1.72 |
| 3 | MCM1_MATALPHA2 | 1 | 0 | 1 | 1.00 | 0.50 | 38.65 |
| 3 | MCM1 | 14 | 11 | 138 | 0.56 | 0.09 | 13.17 |
| 3 | NDT80 | 1 | 0 | 5 | 1.00 | 0.17 | 47.31 |
| 3 | PHO4 | 3 | 1 | 2 | 0.75 | 0.60 | 20.28 |
| 3 | PPR1 | 1 | 0 | 18 | 1.00 | 0.05 | 7.41 |
| 3 | PUT3 | 3 | 0 | 66 | 1.00 | 0.04 | 22.02 |
| 3 | RAP1 | 6 | 16 | 159 | 0.27 | 0.04 | 3.40 |
| 3 | TBP | 7 | 0 | 110 | 1.00 | 0.06 | 30.93 |
| 3 | TFIIA_TBP | 1 | 0 | 1 | 1.00 | 0.50 | 50.66 |
| 3 | TFIIA | 1 | 0 | 6 | 1.00 | 0.14 | -3.55 |
| 4 | GAL4 | 10 | 4 | 98 | 0.71 | 0.09 | 4.10 |
| 4 | GCN4 | 9 | 8 | 146 | 0.53 | 0.06 | 24.23 |
| 4 | HAP1 | 1 | 8 | 0 | 0.11 | 1.00 | 29.70 |
| 4 | LEU3 | 5 | 0 | 2 | 1.00 | 0.71 | 57.58 |
| 4 | MATA1 | 1 | 0 | 22 | 1.00 | 0.04 | 5.74 |
| 4 | MATALPHA2 | 9 | 1 | 142 | 0.90 | 0.06 | 9999.99 |
| 4 | MCM1_MATALPHA2 | 1 | 0 | 2 | 1.00 | 0.33 | 53.40 |
| 4 | MCM1 | 12 | 14 | 122 | 0.46 | 0.09 | 21.72 |
| 4 | NDT80 | 1 | 0 | 13 | 1.00 | 0.07 | 58.85 |
| 4 | PHO4 | 2 | 2 | 0 | 0.50 | 1.00 | 33.71 |
| 4 | PPR1 | 1 | 0 | 51 | 1.00 | 0.02 | 12.77 |
| 4 | PUT3 | 3 | 0 | 111 | 1.00 | 0.03 | 24.67 |
| 4 | RAP1 | 5 | 18 | 174 | 0.22 | 0.03 | 32.81 |
| 4 | TBP | 7 | 0 | 83 | 1.00 | 0.08 | 43.47 |
| 4 | TFIIA_TBP | 1 | 0 | 0 | 1.00 | 1.00 | 66.99 |
| 4 | TFIIA | 1 | 0 | 14 | 1.00 | 0.07 | -2.48 |

Yeast_Self_Reference

| RMSD  Group | Transcription Factor | TP | FN | FP | Sens | Spec | Cutoff |
| --- | --- | --- | --- | --- | --- | --- | --- |
| 0 | GAL4 | 4 | 11 | 0 | 0.27 | 1.00 | -174.36 |
| 0 | GCN4 | 9 | 9 | 4 | 0.50 | 0.69 | -236.36 |
| 0 | HAP1 | 9 | 0 | 95 | 1.00 | 0.09 | -186.96 |
| 0 | LEU3 | 5 | 0 | 3 | 1.00 | 0.62 | -281.51 |
| 0 | MATA1 | 1 | 0 | 0 | 1.00 | 1.00 | -142.86 |
| 0 | MATALPHA2 | 1 | 9 | 0 | 0.10 | 1.00 | -196.74 |
| 0 | MCM1_MATALPHA2 | 1 | 0 | 0 | 1.00 | 1.00 | -354.7 |
| 0 | MCM1 | 1 | 25 | 0 | 0.04 | 1.00 | -190.28 |
| 0 | NDT80 | 1 | 0 | 0 | 1.00 | 1.00 | -288.69 |
| 0 | PHO4 | 3 | 1 | 0 | 0.75 | 1.00 | -247.83 |
| 0 | PPR1 | 1 | 0 | 7 | 1.00 | 0.12 | -143.88 |
| 0 | PUT3 | 3 | 0 | 57 | 1.00 | 0.05 | -124.61 |
| 0 | RAP1 | 19 | 5 | 109 | 0.79 | 0.15 | -298.93 |
| 0 | TBP | 5 | 2 | 2 | 0.71 | 0.71 | -377.94 |
| 0 | TFIIA_TBP | 1 | 0 | 0 | 1.00 | 1.00 | -296.93 |
| 0 | TFIIA | 1 | 0 | 6 | 1.00 | 0.14 | -26.66 |
| 1 | GAL4 | 4 | 11 | 0 | 0.27 | 1.00 | -158.25 |
| 1 | GCN4 | 7 | 11 | 2 | 0.39 | 0.78 | -233.14 |
| 1 | HAP1 | 9 | 0 | 101 | 1.00 | 0.08 | -171.02 |
| 1 | LEU3 | 5 | 0 | 2 | 1.00 | 0.71 | -246.93 |
| 1 | MATA1 | 1 | 0 | 0 | 1.00 | 1.00 | -158.8 |
| 1 | MATALPHA2 | 1 | 9 | 0 | 0.10 | 1.00 | -178.76 |
| 1 | MCM1_MATALPHA2 | 1 | 0 | 0 | 1.00 | 1.00 | -331.67 |
| 1 | MCM1 | 1 | 25 | 0 | 0.04 | 1.00 | -160.62 |
| 1 | NDT80 | 1 | 0 | 0 | 1.00 | 1.00 | -331.55 |
| 1 | PHO4 | 3 | 1 | 0 | 0.75 | 1.00 | -231.34 |
| 1 | PPR1 | 1 | 0 | 8 | 1.00 | 0.11 | -124.64 |
| 1 | PUT3 | 3 | 0 | 9 | 1.00 | 0.25 | -119.64 |
| 1 | RAP1 | 19 | 5 | 103 | 0.79 | 0.16 | -318.58 |
| 1 | TBP | 5 | 2 | 2 | 0.71 | 0.71 | -298.97 |
| 1 | TFIIA_TBP | 1 | 0 | 0 | 1.00 | 1.00 | -216.88 |
| 1 | TFIIA | 1 | 0 | 1 | 1.00 | 0.50 | -28.88 |
| 2 | GAL4 | 3 | 12 | 0 | 0.20 | 1.00 | -182.4 |
| 2 | GCN4 | 7 | 11 | 2 | 0.39 | 0.78 | -249.34 |
| 2 | HAP1 | 8 | 1 | 74 | 0.89 | 0.10 | -191.56 |
| 2 | LEU3 | 5 | 0 | 2 | 1.00 | 0.71 | -255.53 |
| 2 | MATA1 | 1 | 0 | 0 | 1.00 | 1.00 | -173.41 |
| 2 | MATALPHA2 | 1 | 9 | 0 | 0.10 | 1.00 | -173.34 |
| 2 | MCM1_MATALPHA2 | 1 | 0 | 0 | 1.00 | 1.00 | -350.55 |
| 2 | MCM1 | 1 | 25 | 0 | 0.04 | 1.00 | -210.35 |
| 2 | NDT80 | 1 | 0 | 0 | 1.00 | 1.00 | -374.84 |
| 2 | PHO4 | 2 | 2 | 0 | 0.50 | 1.00 | -265.69 |
| 2 | PPR1 | 1 | 0 | 5 | 1.00 | 0.17 | -104.58 |
| 2 | PUT3 | 3 | 0 | 23 | 1.00 | 0.12 | -141.67 |
| 2 | RAP1 | 19 | 5 | 97 | 0.79 | 0.16 | -328.68 |
| 2 | TBP | 2 | 5 | 0 | 0.29 | 1.00 | -298.53 |
| 2 | TFIIA_TBP | 1 | 0 | 0 | 1.00 | 1.00 | -216.96 |
| 2 | TFIIA | 1 | 0 | 1 | 1.00 | 0.50 | -27.63 |
| 3 | GAL4 | 14 | 1 | 28 | 0.93 | 0.33 | -171.04 |
| 3 | GCN4 | 7 | 11 | 2 | 0.39 | 0.78 | -266.83 |
| 3 | HAP1 | 8 | 1 | 61 | 0.89 | 0.12 | -196.2 |
| 3 | LEU3 | 5 | 0 | 2 | 1.00 | 0.71 | -291.52 |
| 3 | MATA1 | 1 | 0 | 1 | 1.00 | 0.50 | -150.69 |
| 3 | MATALPHA2 | 1 | 9 | 0 | 0.10 | 1.00 | -169.32 |
| 3 | MCM1_MATALPHA2 | 1 | 0 | 0 | 1.00 | 1.00 | -318.58 |
| 3 | MCM1 | 21 | 5 | 124 | 0.81 | 0.14 | -124.67 |
| 3 | NDT80 | 1 | 0 | 0 | 1.00 | 1.00 | -468.64 |
| 3 | PHO4 | 2 | 2 | 0 | 0.50 | 1.00 | -250.17 |
| 3 | PPR1 | 1 | 0 | 13 | 1.00 | 0.07 | -88.16 |
| 3 | PUT3 | 3 | 0 | 30 | 1.00 | 0.09 | -136.18 |
| 3 | RAP1 | 18 | 6 | 101 | 0.75 | 0.15 | -325.76 |
| 3 | TBP | 2 | 5 | 0 | 0.29 | 1.00 | -315.94 |
| 3 | TFIIA_TBP | 1 | 0 | 0 | 1.00 | 1.00 | -250.11 |
| 3 | TFIIA | 1 | 0 | 1 | 1.00 | 0.50 | -30.99 |
| 4 | GAL4 | 15 | 0 | 32 | 1.00 | 0.32 | -184.91 |
| 4 | GCN4 | 7 | 11 | 1 | 0.39 | 0.88 | -303.44 |
| 4 | HAP1 | 8 | 1 | 44 | 0.89 | 0.15 | -183.78 |
| 4 | LEU3 | 5 | 0 | 2 | 1.00 | 0.71 | -318.05 |
| 4 | MATA1 | 1 | 0 | 0 | 1.00 | 1.00 | -196.73 |
| 4 | MATALPHA2 | 1 | 9 | 0 | 0.10 | 1.00 | -119.94 |
| 4 | MCM1_MATALPHA2 | 1 | 0 | 0 | 1.00 | 1.00 | -345.08 |
| 4 | MCM1 | 1 | 25 | 0 | 0.04 | 1.00 | -200.88 |
| 4 | NDT80 | 1 | 0 | 0 | 1.00 | 1.00 | -525.24 |
| 4 | PHO4 | 2 | 2 | 0 | 0.50 | 1.00 | -283.37 |
| 4 | PPR1 | 1 | 0 | 26 | 1.00 | 0.04 | -68.98 |
| 4 | PUT3 | 3 | 0 | 25 | 1.00 | 0.11 | -144.66 |
| 4 | RAP1 | 18 | 6 | 108 | 0.75 | 0.14 | -337.19 |
| 4 | TBP | 5 | 2 | 2 | 0.71 | 0.71 | -347.7 |
| 4 | TFIIA_TBP | 1 | 0 | 0 | 1.00 | 1.00 | -279.23 |
| 4 | TFIIA | 1 | 0 | 0 | 1.00 | 1.00 | -42.67 |
