# Supplementary material for: A Structural-Based Strategy for Recognition of Transcription Factor Binding Sites
Source: PLoS One. 2013 Jan 8;8(1):e52460. doi: 10.1371/journal.pone.0052460 (PMC3540023; doi:10.1371/journal.pone.0052460)
Supplement: Table S7 — Prediction Result for Top Ranked ORFs. To suppress the effect of experimental-unknown binding sites and the potential difference between chromosomes, the top ranked ORF numbers were shown in here. These number denotes in each set, for how many ORFs, our lowest energy sequences are overlapping the experimental binding sites. 1. Kono H, Sarai A (1999) Structure-based prediction of DNA target sites by regulatory proteins. Proteins-Structure Function and Genetics 35: 114–131. 2. Robertson TA, Varani G (2007) An all-atom, distance-dependent scoring function for the prediction of protein-DNA interactions from structure. Proteins-Structure Function and Bioinformatics 66: 359–374. (DOC) [file pone.0052460.s007.doc]

**Table S7.** Prediction Result for Top Ranked ORFs

| Training Set | Transcription Factor | Rank Top in the same ORF | | | | |
| --- | --- | --- | --- | --- | --- | --- |
|  |  | RMSD 0 | RMSD 1 | RMSD 2 | RMSD 3 | RMSD 4 |
| Yeast_Self | GAL4 | 12 | 13 | 14 | 14 | 13 |
| Yeast_Self | GCN4 | 8 | 9 | 7 | 7 | 10 |
| Yeast_Self | HAP1 | 3 | 2 | 3 | 3 | 4 |
| Yeast_Self | LEU3 | 5 | 5 | 5 | 5 | 5 |
| Yeast_Self | MATA1 | 1 | 1 | 1 | 1 | 1 |
| Yeast_Self | MATALPHA2 | 6 | 6 | 6 | 6 | 6 |
| Yeast_Self | MCM1_MATALPHA2 | 1 | 1 | 1 | 1 | 1 |
| Yeast_Self | MCM1 | 22 | 17 | 16 | 18 | 18 |
| Yeast_Self | NDT80 | 1 | 1 | 1 | 1 | 1 |
| Yeast_Self | PHO4 | 1 | 1 | 1 | 1 | 1 |
| Yeast_Self | PPR1 | 1 | 1 | 1 | 0 | 0 |
| Yeast_Self | PUT3 | 2 | 1 | 0 | 1 | 1 |
| Yeast_Self | RAP1 | 11 | 11 | 11 | 11 | 10 |
| Yeast_Self | TBP | 4 | 4 | 3 | 3 | 3 |
| Yeast_Self | TFIIA_TBP | 0 | 0 | 0 | 0 | 0 |
| Yeast_Self | TFIIA | 0 | 0 | 0 | 0 | 0 |
| Yeast_Native | GAL4 | 15 | 11 | 4 | 7 | 3 |
| Yeast_Native | GCN4 | 7 | 8 | 2 | 3 | 3 |
| Yeast_Native | HAP1 | 1 | 1 | 1 | 1 | 2 |
| Yeast_Native | LEU3 | 5 | 5 | 3 | 0 | 0 |
| Yeast_Native | MATA1 | 1 | 1 | 1 | 1 | 0 |
| Yeast_Native | MATALPHA2 | 7 | 4 | 3 | 2 | 3 |
| Yeast_Native | MCM1_MATALPHA2 | 1 | 1 | 1 | 0 | 0 |
| Yeast_Native | MCM1 | 20 | 16 | 5 | 2 | 1 |
| Yeast_Native | NDT80 | 1 | 1 | 1 | 0 | 0 |
| Yeast_Native | PHO4 | 1 | 1 | 0 | 0 | 0 |
| Yeast_Native | PPR1 | 1 | 1 | 0 | 0 | 0 |
| Yeast_Native | PUT3 | 2 | 1 | 0 | 0 | 0 |
| Yeast_Native | RAP1 | 12 | 10 | 7 | 10 | 3 |
| Yeast_Native | TBP | 4 | 2 | 0 | 1 | 0 |
| Yeast_Native | TFIIA_TBP | 0 | 1 | 1 | 0 | 0 |
| Yeast_Native | TFIIA | 0 | 0 | 0 | 0 | 0 |
| PDB_Native | GAL4 | 13 | 5 | 0 | 1 | 0 |
| PDB_Native | GCN4 | 0 | 1 | 1 | 0 | 0 |
| PDB_Native | HAP1 | 0 | 0 | 0 | 0 | 0 |
| PDB_Native | LEU3 | 5 | 5 | 0 | 0 | 0 |
| PDB_Native | MATA1 | 0 | 0 | 0 | 0 | 0 |
| PDB_Native | MATALPHA2 | 0 | 0 | 0 | 0 | 1 |
| PDB_Native | MCM1_MATALPHA2 | 1 | 1 | 0 | 0 | 0 |
| PDB_Native | MCM1 | 1 | 0 | 0 | 1 | 0 |
| PDB_Native | NDT80 | 1 | 1 | 1 | 0 | 0 |
| PDB_Native | PHO4 | 1 | 1 | 0 | 0 | 0 |
| PDB_Native | PPR1 | 1 | 1 | 0 | 0 | 0 |
| PDB_Native | PUT3 | 1 | 1 | 0 | 0 | 0 |
| PDB_Native | RAP1 | 3 | 1 | 1 | 0 | 0 |
| PDB_Native | TBP | 0 | 0 | 0 | 0 | 0 |
| PDB_Native | TFIIA_TBP | 0 | 0 | 0 | 0 | 0 |
| PDB_Native | TFIIA | 0 | 0 | 0 | 0 | 0 |
| Yeast_Self_Mutant | GAL4 | 2 | 0 | 1 | 0 | 0 |
| Yeast_Self_Mutant | GCN4 | 0 | 0 | 0 | 0 | 0 |
| Yeast_Self_Mutant | HAP1 | 2 | 2 | 1 | 3 | 1 |
| Yeast_Self_Mutant | LEU3 | 0 | 0 | 0 | 0 | 3 |
| Yeast_Self_Mutant | MATA1 | 0 | 0 | 0 | 0 | 0 |
| Yeast_Self_Mutant | MATALPHA2 | 0 | 0 | 0 | 0 | 0 |
| Yeast_Self_Mutant | MCM1_MATALPHA2 | 0 | 0 | 0 | 0 | 0 |
| Yeast_Self_Mutant | MCM1 | 0 | 1 | 1 | 0 | 0 |
| Yeast_Self_Mutant | NDT80 | 0 | 0 | 0 | 0 | 0 |
| Yeast_Self_Mutant | PHO4 | 0 | 0 | 0 | 1 | 2 |
| Yeast_Self_Mutant | PPR1 | 0 | 0 | 0 | 0 | 0 |
| Yeast_Self_Mutant | PUT3 | 0 | 0 | 0 | 0 | 0 |
| Yeast_Self_Mutant | RAP1 | 0 | 0 | 0 | 0 | 0 |
| Yeast_Self_Mutant | TBP | 0 | 0 | 0 | 0 | 0 |
| Yeast_Self_Mutant | TFIIA_TBP | 0 | 0 | 0 | 0 | 1 |
| Yeast_Self_Mutant | TFIIA | 0 | 0 | 0 | 0 | 0 |
| Yeast_Self_Reference | GAL4 | 6 | 4 | 5 | 5 | 5 |
| Yeast_Self_Reference | GCN4 | 10 | 10 | 8 | 7 | 10 |
| Yeast_Self_Reference | HAP1 | 1 | 1 | 1 | 1 | 0 |
| Yeast_Self_Reference | LEU3 | 4 | 4 | 4 | 4 | 4 |
| Yeast_Self_Reference | MATA1 | 1 | 1 | 1 | 0 | 1 |
| Yeast_Self_Reference | MATALPHA2 | 2 | 2 | 2 | 2 | 2 |
| Yeast_Self_Reference | MCM1_MATALPHA2 | 1 | 1 | 1 | 1 | 1 |
| Yeast_Self_Reference | MCM1 | 7 | 4 | 10 | 9 | 9 |
| Yeast_Self_Reference | NDT80 | 1 | 1 | 1 | 1 | 1 |
| Yeast_Self_Reference | PHO4 | 3 | 3 | 2 | 2 | 2 |
| Yeast_Self_Reference | PPR1 | 0 | 0 | 0 | 0 | 0 |
| Yeast_Self_Reference | PUT3 | 0 | 1 | 0 | 0 | 0 |
| Yeast_Self_Reference | RAP1 | 7 | 8 | 8 | 7 | 6 |
| Yeast_Self_Reference | TBP | 3 | 3 | 3 | 3 | 3 |
| Yeast_Self_Reference | TFIIA_TBP | 1 | 1 | 1 | 1 | 1 |
| Yeast_Self_Reference | TFIIA | 0 | 0 | 0 | 0 | 1 |

To suppress the effect of experimental-unknown binding sites and the potential difference between chromosomes, the top ranked ORF numbers were shown in here. These number denotes in each set, for how many ORFs, our lowest energy sequences are overlapping the experimental binding sites.
