# Supplementary material for: A Structural-Based Strategy for Recognition of Transcription Factor Binding Sites
Source: PLoS One. 2013 Jan 8;8(1):e52460. doi: 10.1371/journal.pone.0052460 (PMC3540023; doi:10.1371/journal.pone.0052460)
Supplement: Text S2 — PBD structures used in decoy tests. (DOC) [file pone.0052460.s009.doc]

**Text S2. PBD structures used in decoy tests**

- 1. DNA sequence decoys

The DNA sequence decoys tests were performed on 51 complexes collected by Kono and Sarai . For each protein-DNA complex, we generate 50,000 evenly distributed random DNA sequences. That is, each base has a probability of 0.25. The DNA structure of a random sequence is constructed by fixing the phosphate-deoxyribose backbone and overlapping the new base pair with the position of the native base pair. The native structures of TF-TFBS are used as templates for threading DNA sequences. In case when there are no native structures of TF-TFBS, For this test, the knowledge functions were trained in a training database of 166 complexes after removing 46 complexes in the dataset of 212 complexes that have higher sequence identity than 35% with the 51 testing complexes. The final training set and testing set list for DNA threading test are:

| Training Set  (PDB id) | 1a0a 1am9 1b01 1bf5 1cez 1cf7 1ckt 1cl8 1cw0 1d02 1dc1 1dct 1dew 1dfm 1diz 1emh 1ewn 1f4k 1fiu 1flo 1fok 1fyl 1gd2 1gu4 1gxp 1h9d 1hlv 1hwt 1i3j 1iaw 1ic8 1j1v 1j3e 1jb7 1je8 1jey 1jj4 1jt0 1jx4 1k3x 1k4t 1ku7 1kx5 1l3l 1lq1 1m3q 1mnn 1mtl 1mus 1odh 1oe4 1omh 1orn 1oup 1p8k 1pp7 1qpi 1qrv 1qzh 1r2z 1r71 1r8d 1r8e 1rh6 1rrq 1rxw 1sa3 1skn 1sxq 1t05 1t9i 1tez 1u3e 1u8b 1uut 1v15 1wb9 1wte 1x9m 1x9n 1xo0 1xpx 1xsd 1xyi 1y8z 1yf3 1z19 1z63 1z9c 1zme 1zs4 1ztw 1zx4 2a3v 2aor 2aq4 2bgw 2bnw 2bsq 2bzf 2c5r 2c7p 2c9l 2dnj 2dp6 2dpi 2dtu 2e1c 2e52 2er8 2ex5 2ezv 2fcc 2fio 2fkc 2fmp 2fr4 2g1p 2gb7 2gig 2h27 2h7g 2heo 2hhv 2ih2 2ihm 2ihn 2is6 2isz 2nq9 2ntc 2o4a 2o8b 2oaa 2odi 2ofi 2owo 2p0j 2p5l 2pyj 2qhb 2qnf 2qsh 2r1j 2r9l 2rba 2rbf 2rgr 2vjv 2vla 2yvh 2z3x 2zhg 3bep 3bkz 3bpy 3brg 3bs1 3btx 3c0w 3c25 3c2i 3clc 3clz 3dvo 3pvi |
| --- | --- |
| Testing Set  (PDB id) | 1a02 1a74 1b3t 1bhm 1bl0 1cdw 1cjg 1cma 1d66 1dp7 1ecr 1fjl 1gat 1gdt 1glu 1hcq 1hcr 1hdd 1hlo 1hry 1if1 1ign 1ihf 1j59 1lmb 1mdy 1mey 1mhd 1mnm 1mse 1oct 1par 1pdn 1per 1pue 1rep 1rv5 1srs 1svc 1tc3 1tf3 1tro 1tsr 1ubd 1xbr 1yrn 1ysa 2bop 2drp 3cro 6cro |

1. Docking Decoys

We obtained near-native docking decoy sets of 45 protein-DNA complexes from Robertson and Varani. There are 2000 lowest-RMSD decoys for each complex generated by FTDock and near-native structures generated from restraints around native complex structures. For this test, a nonhomologous training dataset of 167 complexes is employed (removing 45 complexes in 212 training complexes with sequence identity higher than 35% with these 45 test complexes). The final training set and testing set list for Docking test are:

| Training Set  (PDB id) | 1a0a 1am9 1b01 1bf5 1cez 1cf7 1ckt 1cw0 1dc1 1dct 1dew 1diz 1efa 1egw 1emh 1ewn 1fiu 1flo 1fok 1gd2 1gdt 1gxp 1h6f 1h9d 1i3j 1iaw 1ic8 1j1v 1j3e 1jb7 1jey 1jj4 1jt0 1jx4 1k3x 1k4t 1ku7 1kx5 1m3q 1mdy 1mtl 1mus 1nkp 1odh 1oe4 1omh 1orn 1oup 1owf 1ozj 1p71 1p7h 1p8k 1pp7 1qrv 1qzh 1r2z 1r71 1r8d 1r8e 1rep 1rh6 1rrq 1rxw 1rzr 1sa3 1sxq 1t05 1tez 1u3e 1u8b 1uut 1v15 1w0u 1wb9 1wte 1x9m 1x9n 1xo0 1xpx 1xsd 1xyi 1y8z 1yf3 1z19 1z63 1z9c 1zs4 1ztw 1zx4 2a3v 2ac0 2aor 2aq4 2bgw 2bnw 2bsq 2bzf 2c5r 2c7p 2c9l 2dnj 2dp6 2dpi 2drp 2dtu 2e1c 2e52 2ex5 2ezv 2fcc 2fio 2fkc 2fmp 2fr4 2g1p 2gb7 2gig 2h27 2h7g 2heo 2hhv 2i06 2ih2 2ihm 2ihn 2irf 2is6 2isz 2nq9 2ntc 2o4a 2o8b 2oaa 2odi 2ofi 2owo 2p0j 2p5l 2pyj 2qhb 2qnf 2qsh 2r1j 2r9l 2rba 2rbf 2rgr 2vjv 2vla 2yvh 2z3x 2zhg 3bep 3bkz 3bpy 3brg 3bs1 3btx 3c0w 3c25 3c2i 3clc 3clz 3dfx 3dvo 6cro |
| --- | --- |
| Testing Set  (PDB id) | 1a1i 1a3q 1a73 1au7 1b3t 1bc8 1bdt 1bl0 1ckq 1d02 1dfm 1dmu 1dsz 1eon 1f4k 1fjl 1g9z 1h8a 1hlv 1hwt 1ign 1je8 1jko 1l3l 1lq1 1mjo 1mnn 1pdn 1per 1qna 1qpi 1qpz 1skn 1tc3 1tro 1zme 2bop 2cgp 2dgc 2hdd 3bam 3hts 3pvi 6pax |

c) Dataset for position weight matrices (PWM) prediction test

We use the database of 19 complexes with experimental PWM values collected by Morozov et al. We have removed 1ihf from their original 20-complex set because of the mismatch between the PWM and the DNA bases in the 1ihf complex structure.

For this test, a nonhomologous training dataset of 194 complexes is employed (removing 20 complexes in 212 training complexes with sequence identity higher than 35% with these 19 test complexes). The final training set and testing set list for PWM prediction test are:

| Training Set  (PDB id) | 1a0a 1a3q 1a73 1am9 1b01 1b3t 1bc8 1bdt 1bf5 1bl0 1cez 1cf7 1ckt 1cl8 1cw0 1d02 1dc1 1dct 1dew 1dfm 1diz 1efa 1egw 1emh 1ewn 1f4k 1fiu 1flo 1fok 1fyl 1gd2 1gdt 1gu4 1h6f 1h9d 1hlv 1hwt 1i3j 1iaw 1ic8 1ign 1j3e 1jb7 1je8 1jey 1jj4 1jko 1jt0 1jx4 1k3x 1k4t 1ku7 1kx5 1l3l 1lq1 1m3q 1mdy 1mtl 1mus 1nkp 1odh 1oe4 1omh 1orn 1oup 1owf 1ozj 1p71 1p7h 1p8k 1pp7 1qna 1qpi 1qrv 1qzh 1r2z 1r71 1r8d 1r8e 1rep 1rh6 1rrq 1rxw 1rzr 1sa3 1skn 1sx5 1sxq 1t05 1t9i 1tc3 1tez 1u3e 1u8b 1uut 1v15 1wb9 1wte 1x9m 1x9n 1xo0 1xpx 1xsd 1xyi 1y8z 1yf3 1z19 1z63 1z9c 1zme 1zs4 1ztw 1zx4 2a3v 2ac0 2aor 2aq4 2bgw 2bnw 2bsq 2bzf 2c5r 2c7p 2c9l 2dnj 2dp6 2dpi 2dtu 2e1c 2e52 2er8 2ex5 2ezv 2fcc 2fio 2fkc 2fmp 2fr4 2g1p 2gb7 2gig 2h27 2h7g 2heo 2hhv 2i06 2ih2 2ihm 2ihn 2irf 2is6 2isz 2nq9 2ntc 2o4a 2o8b 2oaa 2odi 2ofi 2owo 2p0j 2p5l 2pyj 2qhb 2qnf 2qsh 2r1j 2r9l 2rba 2rbf 2rgr 2vjv 2vla 2yvh 2z3x 2zhg 3bam 3bep 3bkz 3bpy 3brg 3bs1 3btx 3c0w 3c25 3c2i 3clc 3clz 3coq 3cro 3dfx 3dvo 3pvi 6pax |
| --- | --- |
| Testing Set  (PDB id) | 1aay 1yui 1ysa 1b8i 1fjl 2puc 1yrn 1r0o 1tro 1j1v 2drp 1mj2 1mnn 1gxp 1gcc 1mse 1run 1lmb 6cro |

Our approximate protein-DNA interaction for binding free energies allows the decomposition of the predicted binding free energies into the contributions by each individual base.

In our proposed energy functions, $E_{j}^{i}$ is independent of all other bases. We can calculate position-specific weight matrix (PWM) of a given base *i* at a given position *j* by using the Boltzmann formula:

$$p_{j}^{i}=\frac{\exp(-\beta E_{j}^{i})}{\sum_{i=\{A,C,G,T\}} \exp(-\beta E_{j}^{i})}$$

Where i presents different bases, $\beta=1/RT$is the inverse of temperature and employed as a fitting parameter. The significance of PWM prediction is evaluated by ψ-test. ψ-test is a generalization of well-know $\chi^{2}-test$:

$$\psi(p,q)=\frac{1}{L}\left[\sum_{i=1}^{L} \sum_{\gamma=1}^{4} q_{\gamma}^{i}\ln\frac{q_{\gamma}^{i}}{p_{\gamma}^{i}}\right]$$

where $p_{\gamma}^{i}$ is the predicted probability of base $\gamma$ at position i, $q_{\gamma}^{i}$ is the experimental frequency and L is the length of the binding site in base pairs. To avoid zero denominators, both p and q distributions are smoothed by adding 0.05 to all PWM entries and re-normalizing to avoid zero probabilities at denominator.

1. Gromiha MM, Siebers JG, Selvaraj S, Kono H, Sarai A (2004) Intermolecular and intramolecular readout mechanisms in protein-DNA recognition. Journal of Molecular Biology 337: 285-294.

2. Robertson TA, Varani G (2007) An all-atom, distance-dependent scoring function for the prediction of protein-DNA interactions from structure. Proteins-Structure Function and Bioinformatics 66: 359-374.

3. Morozov AV, Havranek JJ, Baker D, Siggia ED (2005) Protein-DNA binding specificity predictions with structural models. Nucleic Acids Research 33: 5781-5798.
